# Study protocol for a randomised placebo-controlled trial of pramipexole in addition to mood stabilisers for patients with treatment resistant bipolar depression (the PAX-BD study)

**DOI:** 10.1186/s12888-021-03322-y

**Published:** 2021-07-05

**Authors:** Lumbini Azim, Paul Hindmarch, Georgiana Browne, Thomas Chadwick, Emily Clare, Paul Courtney, Lyndsey Dixon, Nichola Duffelen, Tony Fouweather, John R. Geddes, Nicola Goudie, Sandy Harvey, Timea Helter, Eva-Maria Holstein, Garry Martin, Phil Mawson, Jenny McCaffery, Richard Morriss, Judit Simon, Daniel Smith, Paul R. A. Stokes, Jenn Walker, Chris Weetman, Faye Wolstenhulme, Allan H. Young, Stuart Watson, R. Hamish McAllister-Williams

**Affiliations:** 1 Cumbria, Northumberland, Tyne and Wear NHS Foundation Trust, Newcastle, UK; 2 Northern Centre for Mood Disorders, Newcastle University Translational and Clinical Research Institute, Newcastle, UK; 3 Newcastle Clinical Trials Unit, Newcastle University, Newcastle, UK; 4 Biostatistics Research Group, Population Health Sciences Institute, Faculty of Medical Sciences, Newcastle University, Newcastle, UK; 5 Department of Psychiatry, University of Oxford, Oxford, UK; 6 Oxford Health NHS Foundation Trust, Oxford, UK; 7 NIHR Oxford Health Biomedical Research Centre, Oxford, UK; 8 Patient, Carer and Public Involvement, The Newcastle upon Tyne Hospitals NHS Foundation Trust, Newcastle upon Tyne, UK; 9 Clinical Research Network in North East and North Cumbria, Newcastle upon Tyne, UK; 10 Department of Health Economics, Center for Public Health, Medical University of Vienna, Vienna, Austria; 11 Division of Psychiatry and Applied Psychology, University of Nottingham, Nottingham, UK; 12 Institute of Health and Wellbeing, University of Glasgow, Glasgow, UK; 13 Department of Psychological Medicine, Institute of Psychiatry, Psychology and Neuroscience, King’s College London & South London and Maudsley NHS Foundation Trust, Bethlem Royal Hospital, London, UK; 14 Northern Centre for Mood Disorders, Wolfson Research Centre, Campus for Ageing and Vitality, Newcastle upon Tyne, NE4 5LP UK

**Keywords:** Bipolar disorder, Treatment resistant bipolar depression, Pramipexole, Mood stabilisers

## Abstract

**Background:**

Treatment Resistant Bipolar Depression (TRBD) is a major contributor to the burden of disease associated with Bipolar Disorder (BD). Treatment options for people experiencing bipolar depression are limited to three interventions listed by National Institute for Health and Care: lamotrigine, quetiapine and olanzapine, of which the latter two are often not well tolerated. The majority of depressed people with BD are therefore prescribed antidepressants despite limited efficacy. This demonstrates an unmet need for additional interventions. Pramipexole has been shown to improve mood symptoms in animal models of depression, in people with Parkinson’s Disease and two proof of principle trials of pramipexole for people with BD who are currently depressed.

**Methods:**

The PAX-BD study, funded by the United Kingdom (UK) National Institute for Health Research, aims to extend previous findings by assessing the efficacy, safety and health economic impact of pramipexole in addition to mood stabilisers for patients with TRBD. A randomised, double-blind, placebo controlled design is conducted in a naturalistic UK National Health Service setting. An internal pilot study to examine feasibility and acceptability of the study design is included. Participants with TRBD are screened from National Health Service secondary care services in up to 40 mental health trusts in the UK, with the aim of recruiting approximately 414 participants into a pre-randomisation phase to achieve a target of 290 randomised participants. Primary safety and efficacy measures are at 12 weeks following randomisation, with follow up of participants to 52 weeks. The primary outcome is depressive symptoms as measured by Quick Inventory for Depressive Symptomatology – Self Report. Secondary outcomes include changes in anxiety, manic symptoms, tolerability, acceptability, quality of life and cost-effectiveness. Outcome measures are collected remotely using self-report tools implemented online, and observer-rated assessments conducted via telephone. ANCOVA will be used to examine the difference in rating scale scores between treatment arms, and dependent on compliance in completion of weekly self-report measures. A mixed effects linear regression model may also be used to account for repeated measures.

**Trial registration:**

ISRCTN72151939. Registered on 28 August 2019, http://www.isrctn.com/ISRCTN72151939

**Protocol Version:** 04-FEB-2021, Version 9.0.

**Supplementary Information:**

The online version contains supplementary material available at 10.1186/s12888-021-03322-y.

## Background

Bipolar disorder (BD) has a lifetime prevalence of 2.5% [1] and is associated with an 8–12 year reduction in life expectancy [2]. People with BD are symptomatic around 50% of the time, the vast majority of which is depression [3, 4] for which National Institute for Health and Care (NICE) guidelines list 3 treatments: lamotrigine, quetiapine, & olanzapine (with or without fluoxetine) [5]. Quetiapine and olanzapine are often not well tolerated due to weight gain and sedation [6, 7]. In part because of the limited number of medication options, around 70% of people with bipolar depression in the United Kingdom (UK) are taking at least one antidepressant [8] despite evidence that they lack efficacy [5], demonstrating a need for extra therapeutic options.

There is a lack of a consensus definition of what constitutes Treatment Resistant Bipolar Depression (TRBD) [9]. However, around 50% of people remain depressed at six months, and 30% at one year because of non-response, intolerance, or non-acceptance of treatment [10], suggesting that TRBD is the major contributor to the enormous burden of disease associated with BD, with an estimated annual UK BD costs of £5.2B, with direct National Health Service (NHS) costs of £342 M at 2010 prices [11, 12].

The potential role of pramipexole as a treatment for depressive episodes in BD is supported by antidepressant-like effects of pramipexole in animal models such as stress-induced suppression of sucrose intake in rats [13], the forced swim test [14, 15], social interaction test [15] and olfactory bulbectomised rats [16]. It has also been shown to increase hippocampal neurogenesis [12], an effect believed to be common to antidepressants [17, 18]. In humans, pramipexole has extensive evidence for in the treatment of Parkinson’s disease [19], for which it has a marketing licence. A meta-analysis of pramipexole in Parkinson’s disease reported an improvement in depressive symptoms on the Unified Parkinson’s Disease Rating Scale [20]. A subsequent 12-week randomised double-blind placebo-controlled trial of pramipexole in people with Parkinson’s disease and significant depressive symptoms reported a significant benefit independent of any motor improvements seen [21]. Such findings, together with hypothesised roles for a hypo-dopaminergic state underlying bipolar depression [22] and naturalistic and open trial data [23–29] led to two small Randomised Controlled Trials (RCT) in bipolar depression [30, 31]. Goldberg et al studied 22 people with mainly type I BD [30] who had failed to respond to at least two adequate trials of standard antidepressants during the current episode. They were given pramipexole or placebo in combination with lithium or anticonvulsant mood stabilisers, and mean improvement in mood scores at six weeks were 48% for pramipexole and 21% for placebo (*p* = 0.05). Zarate and colleagues’ RCT included 21 people with BD type II [31]who had failed at least one trial of a standard antidepressant. 60% of people treated with pramipexole achieved a treatment response at six weeks, compared with 9% taking placebo (*p* = 0.02).

### Aims

The PAX-BD study is a randomised, double-blind, placebo controlled trial of pramipexole in addition to mood stabilisers for patients with treatment resistant bipolar depression, in a UK NHS population, with the aim of assessing the efficacy and safety of pramipexole and collecting health economic data. PAX-BD is funded by the National Institute for Health Research (NIHR) Health Technology Appraisal (HTA) panel.

### Objectives and measures

A key design feature of the PAX-BD study is that outcome measures are mainly collected remotely, mostly using self-report tools implemented on-line using the True Colours platform [32]. Three scales, the Quick Inventory for Depressive Symptomatology – Self Report (QIDS-SR) [33], Altman Self-rating Scale of Mania (ASRM) [34] scale and Generalised Anxiety Disorder-7 (GAD-7) [35] scale are completed on-line by participants weekly from recruitment into the pre-randomisation phase through to the end of follow up. To facilitate comparison of PAX-BD with other studies, the clinician-rated version of the QIDS (QIDS-C) [33], the Montgomery-Asberg Depression Rating Scale (MADRS) [36] and the Young Mania Rating Scale (YMRS) [37] are completed by phone by the study Research Assistants (RAs) at two time points. The timings of the administration of assessments is shown in the Schedule of Events (Table 1).

**Table 1:** Schedule of Events

	Screening	Pre-randomisation	Screening and Randomisation	0	Treatment weeks (Post-randomisation)																												Tapering (5, 6, 7)	
					1	2	3	4	5	6	7	8	9 to 11	12	13 to 15	16	17 to 19	20	21 to 23	24	25 to 27	28	29 to 31	32	33 to 35	36	37 to 39	40	41 to 43	44	45 to 47	48	49 to 51	52*
Informed consent (1,2)	✓		✓																															
Demographics	✓	✓																																
Eligibility assessment	✓		✓																															
Randomisation			✓																															
RA safety monitoring call (3, 8)		✓		✓	✓	✓	✓	✓	✓	✓	✓	✓	✓	✓		✓		✓		✓		✓		✓		✓		✓		✓		✓	✓	✓
CSO contact (4)						✓				✓				✓						✓						✓						✓		✓
QIDS-SR, GAD-7 and ASRM		✓		✓	✓	✓	✓	✓	✓	✓	✓	✓	✓	✓	✓	✓	✓	✓	✓	✓	✓	✓	✓	✓	✓	✓	✓	✓	✓	✓	✓	✓	✓	✓
SHAPS				✓						✓				✓																				
WSAS				✓						✓				✓						✓						✓						✓		
TSQM										✓				✓		✓		✓		✓		✓		✓		✓		✓		✓		✓		
QUIP-RS				✓						✓				✓		✓		✓		✓		✓		✓		✓		✓		✓		✓		✓
MADRS, QIDS-C and YMRS				✓										✓																				
IMP Dispensings			✓					✓						✓				✓				✓				✓				✓				
Qualitative Interviews		✓												✓																				
Participant Vouchers														✓												✓								✓
Unblinding (9)																																✓		

^[1]^Consent is received to enter the pre-randomisation phase. ^2^ To be randomised to trial medication. ^**3**^ Weekly through pre-randomisation phase and until participant begins trial medication. ^**4**^ CSO or other delegated person at site – this will involve collection of medication returns and urine samples. ^5^ If a participant stops taking medication for any reason during the trial, RA phone calls including Dopamine Agonist Withdrawal Syndrome screening will take place weekly during tapering. If participant has withdrawn from the trial, final safety assessment including pregnancy test for women of child-bearing potential will take place when participant has been drug-free for 2 weeks. ^6^ Final safety assessments will take place at week 52 or when participant has been drug-free for 2 weeks, whichever is later. ^7^Week 49–52 schedule of assessments for tapering not applicable for participants who have been unblinded and are taking placebo. Participants receiving placebo will receive a final ‘thank you’ RA phone call following unblinding medication. No further safety assessments, including pregnancy test, will be undertaken for these participants. ^**8**^ Weekly through tapering phase. ^9^ Unblinding to take place after week 48 assessments only for participants who have indicated that they would wish to continue taking pramipexole after the end of the trial, if they were found to be receiving it. *Plus up to 2 weeks if required

### Primary objective

- To evaluate the clinical effectiveness of pramipexole versus placebo alongside standard mood stabilising medication, over 12 weeks, in the management of participants with treatment resistant bipolar depression, assessed using the QIDS-SR [33].

### Secondary efficacy objectives

- To examine the impact of pramipexole treatment on mood and anxiety symptoms over 48 weeks, and pleasure symptoms over 12 weeks. In addition to the weekly assessment of depressive and anxiety symptoms with the QIDS-SR and GAD-7 [33, 35] respectively, the ability to experience pleasure is assessed using the Snaith-Hamilton Pleasure Scale (SHAPS) [38] at randomisation and at weeks 6 and 12 post-randomisation.
- To examine the impact of pramipexole on psychosocial function over 48 weeks, using the self-reported Work and Social Adjustment Scale (WSAS) [39].

### Secondary safety and acceptability objectives

- To examine risk of switching to mania and occurrence of psychosis or impulse control disorders, which are known possible side-effects of pramipexole [24], over 48 weeks. Manic symptoms are assessed weekly using an on-line version of the ASRM scale [34]. Rates of impulsivity during the study are monitored using the Questionnaire for Impulsive-Compulsive Disorders in Parkinson’s disease – Rating Scale (QUIP-RS) [40], administered by telephone by the study RAs. Psychosis is not formally rated but is specifically screened for during regular RA phone calls.
- To examine side effects and overall acceptability of pramipexole treatment over 48 weeks using the Treatment Satisfaction Questionnaire for Medication (TSQM) [41] administered by phone by the study RAs.
- To examine tolerability of pramipexole by reviewing the rates, severity, seriousness, causality and expectedness Adverse Events (AEs) and Adverse Reactions (ARs).
- To examine adherence to medication over 48 weeks. Medication is provided to participants by post from the Cumbria, Northumberland, Tyne and Wear (CNTW) pharmacy on seven occasions over the course of the study. Adherence is assessed through a pill count of un-used medication returned by participants at the end of each prescription period.

### Health economic objectives

- To examine the quality of life, wellbeing, health and social care and broader societal costs of participants randomised to either pramipexole or placebo. To establish the incremental cost-effectiveness of pramipexole in comparison to placebo over 48 weeks. Assessments are conducted via participant completion of on-line versions of the EuroQoL 5 Dimension 5 Level (EQ-5D-5L) [42] measure of health-related quality of life, and the ICEpop CAPability measure for Adults (ICECAP-A) [43] and the Oxford CAPabilities questionnaire-Mental Health (OxCAP-MH) [44] capturing broader wellbeing, and the Health Economics Questionnaire (HEQ) [45] for information on health and social services utilisation and broader societal costs.

#### Qualitative interviews

Qualitative interviews are undertaken during the internal pilot to explore variables impacting recruitment, retention rates and discontinuation. The Topic Guide is included in Supplementary file 12. Interviews are administered by phone to staff in sites open for recruitment for at least 4 weeks, participants who withdraw/are withdrawn during the pre-randomisation phase or before week 12 in the randomisation phase, and randomised participants who remain in the trial until week 12. Interviews will conclude for each group once data saturation has been reached.

### Sample size, power and effect size

The estimated sample size for randomisation is based on the CEQUEL study [32] that included similar participants in a depressive episode of BD from the UK NHS, and who had weekly remote monitoring of mood over a 52 week period. Dropout rates in this study were 20% at 12 weeks and 50% at 52 weeks. A power calculation demonstrates that 290 participants provides a 90% power of detecting a 3-point difference in QIDS-SR [33] between pramipexole and placebo (*p* < 0.05), based on a two-sample t-test at 12 weeks with a QIDS-SR [33] standard deviation of 7 and assuming a 20% dropout rate as seen in the CEQUEL study [32]. This sample size also provides approximately 80% power of detecting a 3.3 point difference in QIDS-SR scores [33] between the treatment arms at week 48 assuming the 50% dropout rate seen in CEQUEL [32] and the standard deviation used above. In a study of a similar population, a drop-out rate of 30% was experienced during the pre-randomisation phase [46]. As a result, it is anticipated that PAX-BD will need to recruit approximately 414 participants into the pre-randomisation phase trial to achieve the target of 290 participants randomised.

## Methods

### Study design

The PAX-BD study is a multicentre parallel group, double-blind, randomised, placebo-controlled superiority trial of the addition of pramipexole to ongoing mood stabiliser (lithium, valproate, carbamazepine or lamotrigine) treatment in patients suffering from a moderate to severe episode of bipolar depression who are operationally defined as having TRBD on the basis of non-response, intolerance, contraindication, clinically felt to be inappropriate or declined by patient of at least two NICE recommended treatments for bipolar depression (lamotrigine, quetiapine, olanzapine with or without fluoxetine), or lurasidone which is included as a recommended treatment in British Association for Psychopharmacology guidelines [47].

The study was initially funded and approved on the basis that an exclusion criterion for randomisation was ongoing treatment with an antipsychotic. Those participants on antipsychotics recruited into stage 1, were required to have these withdrawn. Early feedback during the study pilot phase indicated that this requirement was significantly negatively impacting on recruitment. As a result, this issue was reviewed. Since the original submission of the study design to the funder, increasing evidence has emerged suggesting that pramipexole’s mechanism of action in depression is via activation of dopamine D3 receptors. Pharmacologically, pramipexole appears to preferentially bind to D3 versus D2 receptors in vivo [48]. D3 receptor knock out mice have been reported to exhibit depressive and anxious features [49]. In addition, a wealth of recent data using highly selective D3 antagonists in animal models has demonstrated that D3 receptors play a critical role in reward processes [50, 51]. Further, PET imaging data in humans suggests D3 receptor expression may be related to motivation for rewards [52]. Recent data also suggests a positive effect of pramipexole on dendritic arborization and soma size mediated via increased brain derived neurotrophic factor (BDNF: 55), and that pramipexole can prevent inflammation induced depression-like behaviours in mice [53], both plausible mechanism of action in depression. On this basis, the HTA panel and the Medicines and Healthcare products Regulatory Agency (MHRA) approved an alteration to the protocol to allow participants to be on an antipsychotic at randomisation if the dose was at or below the level described in Table 2. These doses were determined based on the individual drug’s absolute D3 receptor affinity and the relative affinity for D2 versus D3 receptors.

**Table 2:** List of antipsychotics and maximum daily dose allowed for randomisation eligibility

Drug	Maximum daily dose
Aripiprazole	15 mg
Aripiprazole depot	400 mg every 4 weeks
Chlorpromazine	200 mg
Flupentixol depot	200 mg every 4 weeks
Haloperidol	2 mg
Haloperidol depot	100 mg every 4 weeks
Lurasidone	111 mg
Olanzapine	10 mg
Olanzapine depot	150 mg every 2 weeks
Paliperidone	3 mg
Paliperidone depot	75 mg every month
Quetiapine	300 mg
Risperidone	1 mg
Risperidone depot	25 mg every 2 weeks
Zuclopenthixol depot	500 mg every 4 weeks

The study has two phases: a pre-randomisation phase and a post-randomisation phase. The pre-randomisation phase is to allow participants to adjust antipsychotic medication to minimise any possible antagonism of the effect of pramipexole and commence mood stabilisers, if needed; these doses must be stable for a minimum of four weeks prior to randomisation. If no changes to medication are required, the pre-randomisation phase lasts for four weeks from consent to randomisation to allow familiarisation with the data collection tools and confirm persistent depressive symptoms. The primary outcome point is 12 weeks after randomisation, with follow up of participants to 48 weeks, plus 4–6 weeks to allow for continuing or tapering of trial medication.

PAX-BD includes an internal pilot study to examine feasibility and acceptability of the study design and inform any remedial action to the study design if required. Qualitative and quantitative data is collected from participants during the pre-randomisation phase and up to 12 weeks post randomisation, along with qualitative interviews of research staff regarding recruitment and management of participants in the study. The trial funder stop criteria are, relative to recruitment of the first participant into the pre-randomisation phase of the trial, ≤ 50 participants randomised to either arm at 12 months with a ≤ 70% retention of those randomised at the primary outcome time point of 12 weeks. At 12 months post-consent to pre-randomisation of the first participant, the Trial Management Group (TMG) will review the number of participants randomised and the percentage retention at 12 weeks. These numbers will be passed to the Data Monitoring Committee (DMC) and Trial Steering Committee (TSC), for verification as to whether they meet the trial stopping criteria or not. The TSC monitor study progress and conduct, and an independent DMC are the only body with access to un-blinded data prior to the final data lock at study conclusion. Recruitment and retention rates will be reviewed on an ongoing basis by the TMG throughout the trial.

### Participants

#### Recruitment

Patients with TRBD are screened and recruited from secondary care services using clinician caseloads, databases and/or research registers in up to 40 mental health trusts across the UK. A list of study sites can be obtained at https://paxbd.org/about/recruiting-sites. Invitation letters and summary leaflets (Supplementary files 1 and 2) are available to advertise to patients directly by post, or channels such as secondary care clinics, websites, social media platforms and patient support groups.

Patients identified by clinicians and/or those showing interest are provided an initial information sheet (Supplementary file 3) in person or via post. Should they wish to proceed, Principal Investigators (PIs) or delegated clinicians take written informed consent (Supplementary file 4) and begin the screening process. Prior to randomisation participants receive a second information sheet (Supplementary file 5) and again are required to provide written informed consent (Supplementary file 6).

**Fig. 1:**
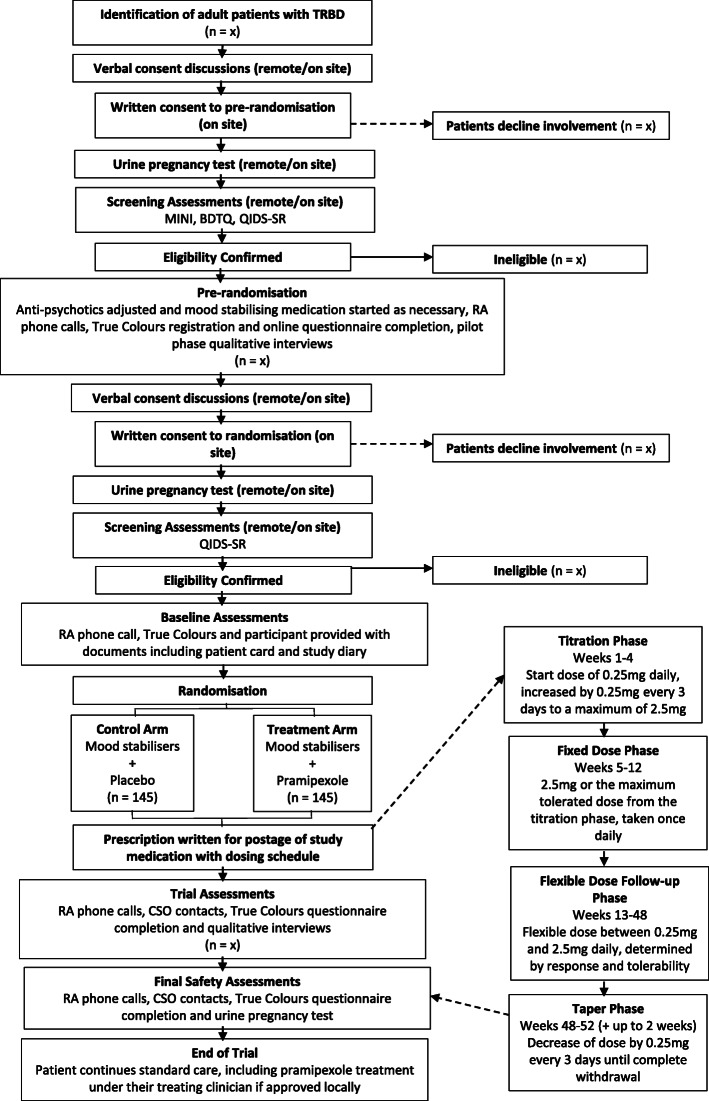
Participant Pathway

Participants who withdraw from the trial are given a withdrawal form (Supplementary file 7) and corresponding end of study information sheet (Supplementary file 8). Withdrawn participants are not replaced. Where a participant no longer wishes to take trial medication and gives consent for continued participation in the trial, they are followed up to 48 weeks. Participants who complete the trial to 48 weeks with trial medication are given the corresponding end of study information sheet (Supplementary file 9). See Fig. 1 for the participant pathway.

#### Inclusion criteria for entry to the pre-randomisation phase

- Currently under the care of secondary care mental health services at screening with a plan for the patient to remain in secondary care throughout the period of the trial.
- A decision made by the patient’s clinical team that a change in medication is indicated.
- A current diagnosis of Bipolar Disorder (type I or II), defined as in Diagnostic and Statistical Manual of Mental Disorders-5 (DSM-5) [54], which is supported by the use of the Mini-International Neuropsychiatric Interview (MINI) [55].
- Currently depressed, i.e. meeting DSM-5 criteria for a Major Depressive Episode assessed via MINI and with a current Quick Inventory of Depressive Symptomology – Self Report (QIDS-SR) > 10 [33, 54].
- Current episode of depression failed to have responded to adequate trials, or lack of tolerability or patient declined/clinically inappropriate, of two different NICE recommended medications (quetiapine, olanzapine with or without fluoxetine, lamotrigine) or lurasidone. Adequacy of treatment trial defined using a custom designed ‘Bipolar Demographics and Treatment Questionnaire’ (BDTQ).
- Aged 18 or over at the point of consent.
- Willing and able to provide written informed consent prior to any trial procedures taking place.
- In the opinion of the investigator, is able to follow the trial prescription instructions and is able to manage 8 weeks supply of trial medication without risk of overdose.
- The patient, if female and of child-bearing potential, must have a negative pregnancy test [urine beta-human chorionic gonadotropin (β-hCG)].
- Women of child-bearing potential are required to use a highly effective contraceptive method during the pre-randomisation and post-randomisation phase of the trial. Highly effective methods of contraception include:
  - combined (oestrogen and progestogen containing) hormonal contraception associated with inhibition of ovulation (oral, intravaginal, transdermal)
  - progestogen only hormonal contraception associated with inhibition of ovulation (oral, injectable, implantable)
  - intrauterine device
  - intrauterine hormone-releasing system
  - vasectomised partner (provided that partner is the sole sexual partner of the trial participant and that the vasectomised partner has received medical assessment of the surgical success)
  - bilateral tubal occlusion
  - sexual abstinence (defined as refraining from heterosexual intercourse during the entire period of risk associated with the study treatments. The reliability of sexual abstinence needs to be evaluated in relation to the duration of the clinical trial and the preferred and usual lifestyle of the subject)

#### Exclusion criteria for the pre-randomisation phase

- DSM-5 defined severe substance use disorder.
- Current psychotic symptoms as assessed using the MINI.
- History of retinal disease.
- Current cardiovascular symptoms or significant concerns around cardiovascular disease.
- History of significant renal disease (for example within the last 6 months estimated Glomerular Filtration Rate (eGFR) is less than 50 ml/min/1.73 m2 or there is a concern that eGFR is deteriorating and may be expected to fall below 50 during the course of the study).
- Any known sensitivity to trial drug including its excipients.
- Current pregnancy or planned pregnancy during the trial period, or breastfeeding.
- Starting specific psychotherapy from four weeks before randomisation through to Week 12 post-randomisation.
- Currently taking part in another clinical trial that would interfere with the outcomes of PAX-BD.
- Confirmed diagnosis with potential confounding factors such as Parkinson’s disease, restless leg syndrome (where restless legs syndrome has been formally diagnosed by a sleep clinic).
- Significant clinical concern regarding impulse control behaviours.

#### Inclusion for randomisation

All criteria as for the pre-randomisation plus:

- Been in pre-randomisation for a minimum of 23 calendar days.
- Severity of depression still meeting the criteria of QIDS-SR > 10.
- A minimum of two telephone phone calls with a trial RA and two on-line weekly symptom ratings have been completed during the pre-randomisation phase
- On mood stabilising medication (lithium, valproate, carbamazepine, lamotrigine)
- If on an antipsychotic this must be one listed, and at a dose of no more than the maximum stated in Table 2.
- All regular psychotropic medication, including antipsychotics and mood stabilisers, at a stable dose for a minimum of four weeks. Additionally, if a participant is on lamotrigine, quetiapine, olanzapine or lurasidone then this must have been at the current dose or higher for a minimum of three months.

#### Exclusion criteria for randomisation

As for the pre-randomisation phase plus:

- Psychotic symptoms over the preceding 4 weeks.
- Any deterioration in physical or mental health since pre-randomisation that means there is a clinical concern to proceed with the study.
- Any study team’s concern regarding the patient’s ability to remain engaged in the study collecting self-ratings of their symptoms.

### Intervention

Participants are randomised on a 1:1 basis to receive either pramipexole or placebo. Dosing of study medication is based on doses used in the two previous small pilot studies (*n* = 22; *n* = 21) [30, 31] and, in particular, a naturalistic case series [24]. Dosing occurs in three phases: titration, fixed dose and flexible dose phases. During the titration phase, participants start on a dose of 0.25 mg/daily with 0.25 mg increments every three days to reach the maximum tolerated dose, with a maximum of 2.5 mg/daily, over four weeks. The dose achieved in the titration phase is continued through the fixed-dose phase between weeks 5 to 12 post randomisation. From week 12 to the end of the study, medication is flexibly dosed between 0.25 and 2.5 mg/day, based on the participant’s trajectory of change in mood and tolerability as judged by self-reported measures and side effects.

At 48 weeks, remaining participants are asked whether they wish to continue with pramipexole after the trial if they are found to be taking it; those who do are unblinded to find if they were receiving the drug or placebo. This allows for the immediate withdrawal of participants on placebo, and the continuation of treatment for those receiving pramipexole beyond the study. Participants who wish to discontinue study medication are not unblinded and receive a tapering schedule of a reduction by 0.25 mg every three days, to safely withdraw from trial medication.

Participant’s clinical teams are free to adjust all other medication, though encouraged wherever possible to maintain stability of all medication and doses between randomisation and the 12 week primary outcome point. Participants are provided study diaries at the start of randomisation to guide them through the titration phase, record the dose of medication taken each day, record any changes to concomitant medication and record AEs (Supplementary files 10 and 11). Adherence to the dose schedule is verbally checked with the participant during RA telephone calls.

AEs are recorded by trial RAs for all participants from the date of consent to the pre-randomisation phase until the participant’s final trial assessment, or until participant withdraws. AEs are recorded on the trial’s MACRO™ system by the trial RAs. Line listings of all AEs for a participant are sent to the site PI or delegated clinician for assessment of seriousness, severity and causality. Serious Adverse Events must be reported within 24-h by all staff members as soon as they become aware of the event.

#### Randomisation and blinding

Sealed Envelope, a secure web-based system managed centrally is used to randomise participants with concealed allocation. The first 10 participants are allocated randomly. Subsequently, a non-deterministic minimisation algorithm is used with an 80% bias in favour of allocations that minimise an imbalance on variables hypothesised to be related to prognosis at baseline: bipolar I or bipolar II based on DSM-5 criteria; severity of depression at randomisation (moderate, severe or very severe); age (18–50 or > 50); biological sex (male or female); UK site region (North, Midlands and East, London, South East, South West, Scotland, Wales or Northern Ireland); concurrent mood stabiliser (lithium, valproate, lamotrigine, carbamazepine or multiple mood stabilisers); concurrent antidepressant (yes or no); on an antipsychotic at randomisation (yes or no); number of mood episodes in the past year (< 4 or ≥ 4).

Pramipexole and matching placebo are manufactured by Wasdell then packaged and labelled by Modepharma. Trial medication is then delivered blinded to the CNTW pharmacy for storage and dispensing. The TMG is blind to treatment allocations. An unblinded Data Manager will provide the DMC the key to unblind treatment arms for closed reports. To prepare these reports, a trial statistician is partially blinded and provided data by the unblinded Data Manager where treatment arms are coded as A and B. Participants and staff at recruitment sites remain blinded for the duration of the study, though unblinding can occur in the event of a clinical emergency.

### Data handling

Data is handled, computerised and stored in accordance with the Data Protection Act 2018. Paper copies of trial related documentation are annotated, signed and dated, and filed in the medical notes. The overall quality and retention of trial data is the responsibility of the Chief Investigator (CI). All trial data is retained in accordance with the latest Directive on GCP (2005/28/EC) and local policy.

Clinical and safety data for trial participants is collected by site staff and trial RAs, and recorded in the electronic case report form (eCRF) of the clinical data management system MACRO™ and password protected trial spreadsheets. Unique trial identifier numbers are used for participant identification on the eCRFs and trial spreadsheets.

Primary outcome data, in the form of answers to all trial questionnaires (Health Economic and otherwise) are entered directly by the trial participants into the University of Oxford True Colours online platform. Participants receive login details in the form of a username and password at the start of the trial, and change their password to something only they know.

### Statistical analysis

Full details of all statistical analyses will be specified in a pre-defined Statistical Analysis Plan. Initial descriptive analysis will present the profile of the participants and investigate differences in demographic and outcome measures between the two treatment arms at baseline. If significant differences are found, the variable may be added to the models testing the relevant study hypothesis. A two-sided significance level of *p* < 0.05 will be used throughout.

Analysis of the primary outcome measure of QIDS-SR [33] at week 12 will use Analysis of Covariance (ANCOVA) to examine the difference between the treatment arms with adjustment for baseline covariates including initial QIDS-SR score, other minimisation factors and anxiety (GAD-7) score [35]. Other baseline covariates will be examined for potential inclusion during the modelling process.

Weekly repeated measures scores for QIDS-SR [33] will be examined to ascertain participant compliance in completion. Should this be judged sufficient, a mixed effects linear regression model will be used to account for the repeated measures over time in comparing this outcome between the treatment groups. Other secondary outcome measures include: the proportion of participants in remission at 12 and 48 weeks (QIDS-SR score ≤ 5), the proportion of participants who have achieved a response at 12 and 48 weeks (QIDS-SR score reduction of ≥50% from baseline), proportion of time over 48 weeks that participants are free of depressive symptoms (QIDS-SR score ≤ 5), proportion of time over 48 weeks that participants are free of manic symptoms (ASRM score ≤ 5) and changes in psychosocial function determined by the WSAS (35;37;42).

Secondary outcomes, other than those collected specifically for the health economic analysis, will be analysed in a similar manner to the primary outcome as described above. The ASRM and GAD-7 scores will also be examined on a weekly basis as described for the QIDS-SR [33–35]. MADRS, YMRS, SHAPS and QIDS-C between 0 and 12 weeks will be examined in a manner analogous to the primary analysis of the primary outcome [33, 36–38]. Safety and tolerability assessments will include ANCOVA of TSQM and QUIP-RS scores [40, 41], co-varying for baseline score and GAD-7 anxiety scores [35] given the impact of anxiety on tolerability and reported side effects with medication.

Levels of missing data will be described and baseline values tabulated for those for whom the primary end point can and cannot be calculated in order to summarise any characteristics related to missingness. Data with missing observations due to participant withdrawal or loss to follow-up will be examined to determine both its extent and whether it is missing at random. It is likely that missingness will be related to the outcome itself with depressed participants less likely to complete assessments. However, it has been suggested that even when non-random, a valid approach is to assume randomness since results tend to remain stable when randomness is violated. The primary analysis will utilise complete case analysis with covariate adjustment. This yields similar results to multiple imputation in data that are missing randomly, as long as predictors of missingness are included. If data are missing to a sufficient extent (e.g. between approximately 10 and 20%), the use of appropriate multiple imputation techniques will be considered. The QIDS-SR [33] is assessed weekly. For the primary analysis, the assessment closest to 12 weeks in the range 10–14 weeks will be employed. Missing items from a partially completed TQSM questionnaire [41] will be handled as described in the scoring manual.

#### Health economic analysis

The main health economic analysis will include a detailed patient-level cost analysis of health, social care and other broader societal costs for both the pramipexole and placebo arms of the study, and an incremental within-trial economic evaluation comparing the pramipexole and placebo arms of the trial in terms of their costs and outcomes over the 48 weeks trial follow-up period.

The cost analysis will be based on resource use data collected as part of a bespoke HEQ [45]; collected data will include all hospital and community health and social services, medication, productivity losses, informal care and patient’s travel expenses. Costing will be conducted using national-level unit costs from the UK for a common year, e.g. Personal Social Services Research Unit Costs Database [56] and British National Formulary [57]. Lost productivity costs due to absenteeism or presenteeism will be estimated using the human capital approach where time off work is multiplied by the average daily national salary for participants who are employed or self-employed [58, 59].

The primary health economic analysis will be a cost-utility analysis from a health and social care perspective where quality-adjusted life years will be calculated using utility values from the EQ-5D-5L [42] health-related quality of life questionnaire as recommended by most health technology assessment agencies [60–62]. Health states will be valued by using the latest valid tariff set from the UK and results will be expressed in an incremental cost-effectiveness ratio (ICER).

Secondary economic analyses using the ICECAP-A (46) and the OxCAP-MH [44] capability indices as outcome measures will be also carried out. The capability states measured by the ICECAP-A will be valued by the tariff set for the UK [63]. A similar tariff weighting score concept will be developed for the OxCAP-MH [44] alongside this trial and will subsequently be applied in the valuation of the dataset. Further analyses will estimate cost-effectiveness from a societal perspective.

Multiple imputation will be used to deal with missing data. Results from both the full imputed dataset and from the complete case analysis will be compared and reported as means with standard deviations or as mean differences with 95% confidence intervals. Differences in mean costs and effects will be compared in a regression framework with a *p*-value less than 5% considered as statistically significant. Non-parametric bootstrapping [64] from the cost and effectiveness data will be used to generate a joint distribution of the mean incremental costs and effects for the options under comparison and to calculate the 95% confidence intervals of the ICERs. Uncertainty around the main cost-effectiveness estimates will be represented by cost-effectiveness acceptability curves (CEACs) using the net benefit approach [65, 66]. CEACs show the probability that each option is cost-effective to a range of maximum values (ceiling ratio) that a decision maker might be willing to pay for an additional unit of improvement in outcomes.

#### Qualitative analysis

The qualitative analysis will be based on interviews with participants and medical personnel at sites. Analysis of barriers and facilitators will specifically draw on a meta-analysis of studies in depression, providing a framework for the qualitative interview. Interviews will be transcribed and analysed according to the framework as data is collected; collection will conclude once the data is saturated.

### Status of the study

In light of the COVID-19 UK lockdown, recruitment to the study was paused and two participants in the pre-randomisation phase were withdrawn. One participant had progressed to the randomisation phase and remained in the study. Substantial Amendment 7 was initiated to accommodate necessary changes and allow greater flexibility for face to face study activities in response to the COVID-19 pandemic – the amendments are detailed in Supplementary file 13. The PAX-BD study re-opened for recruitment on 15th September 2020. A key further amendment to the study protocol was Substantial Amendment 11, which altered the eligibility criteria at the randomisation stage to allow for participants to be included who are on antipsychotics as listed in Table 2. This amendment was enacted on 9th April 2021. At this point, five participants had entered stage 2 of the study prior to the amendment.

## Discussion

PAX-BD will be the first large adequately powered RCT in a well-defined population of patients with TRBD. The study not only examines the efficacy, tolerability and safety of pramipexole, but also its cost effectiveness over a prolonged follow up period of one year. If pramipexole is shown to be safe and (cost) effective, then it could be made available to patients rapidly given that it is a currently licenced mediation, albeit for other indications. The PAX-BD study will provide significant information to help guide clinicians in the use of the drug. A “clinician manual” has been prepared for clinicians managing participants in the study. This will be iteratively updated during the course of the study and made available on completion of the study.

The design of the study is an evolution from two previous large RCTs in BD run in the UK NHS – BALANCE [46] and CEQUEL [32]. PAX-BD extends the extent to which data is self-reported by participants and collected remotely. This has many advantages including the ability to run the study from a central base with minimal need for direct input from clinical teams in participating sites. This is an important consideration given the difficulty of hard-pressed clinicians actively engaging in research. In addition, just as recruitment was commencing, the COVID-19 pandemic occurred. The design of PAX-BD enables minimal face to face contact and hence reduced infection risks for participants and study staff. The qualitative data collected during the study will help inform if the PAX-BD design is effective and how study and clinical staff view it. This will help inform the design of future studies.

### Dissemination policy

Dissemination to the academic community will include a final report for the funders, as well as findings presented at international scientific meetings and submitted for publication in high-impact open-access peer-reviewed journals. Dissemination to clinicians will include web-based information on the Northern Centre for Mood Disorders (NCMD) [67] and PAX-BD study [68] websites, with links to this from all Universities and Trusts involved in the trial and verbal presentations at meetings such as those organised by the British Association of Psychopharmacology (BAP) and the Royal College of Psychiatrists. The research findings will be disseminated to Clinical Commissioning Groups via the CI and PI links as clinicians within regionally facing specialist mood disorders services. The findings will be supplied to the two main pharmacological treatment guidelines used by clinicians in the UK: those produced by NICE and the BAP.

The trial results will be disseminated to patients and carers in partnership with the trial patient advisory group. A leaflet explaining the findings of the trial and their implications will be produced alongside two-monthly public engagement meetings advertised via the NCMD website to promote and disseminate materials. Dissemination will also be via the patient organisation Bipolar UK and Bipolar Scotland and to the general public e.g. via the Science Media Centre. The results will also be disseminated to the Drug and Therapeutics (or equivalent) Committee of each participating Trust via the local PIs.

## Supplementary Information

**Additional file 1.**

**Additional file 2.**

**Additional file 3.**

**Additional file 4.**

**Additional file 5.**

**Additional file 6.**

**Additional file 7.**

**Additional file 8.**

**Additional file 9.**

**Additional file 10.**

**Additional file 11.**

**Additional file 12.**

**Additional file 13.**

## Abbreviations

AE: Adverse Event
ANCOVA: Analysis of Covariance
AR: Adverse Reaction
ASRM: Altman Self Rating Scale of Mania
BAP: British Association of Psychopharmacology
BD: Bipolar Disorder
BDNF: Brain derived neurotropic factor
BDTQ: Bipolar Demographics and Treatment Questionnaire;
CI: Chief Investigator
CNTW: Cumbria, Northumberland, Tyne and Wear
DMC: Data Monitoring Committee
DSM-5: Diagnostic and Statistical Manual of Mental Disorders, Fifth Edition
eCRF: Electronic Case Report Form;
eGFR: Estimated glomerular filtration rate
EQ-5D-5L: EuroQoL 5 Dimension 5 Level
HEQ: Health Economics Questionnaire
HTA: Heath Technology Assessment
ICER: Incremental Cost-Effectiveness Ratios
MADRS: Montgomery Asberg Depression Rating Scale
MHRA: Medicines and Healthcare products Regulatory Agency
MINI: Mini-International Neuropsychiatric Interview
NCMD: Northern Centre for Mood Disorders
NHS: National Health Service
NICE: National Institute for Health and Care Excellence
NIHR: National Institute of Health Research
OxCAP-MH: Oxford CAPabilities Questionnaire – Mental Health
PI: Principal Investigator
QIDS-C: Quick Inventory of Depressive Symptomatology – Clinician Rated
QIDS-SR: Quick Inventory of Depressive Symptomatology – Self Report
QUIP-RS: Questionnaire for Impulsive-Compulsive Disorders in Parkinson’s Disease – Rating Scale
RA: Research Assistant
RCT: Randomised Control Trial
SHAPS: Snaith-Hamilton Pleasure Scale
TMG: Trial Management Group
TSC: Trial Steering Committee
TSQM: Treatment Satisfaction Questionnaire for Medication
TRBD: Treatment Resistant Bipolar Depression
UK: United Kingdom
WSAS: Work and Social Adjustment Scale
YMRS: Young Mania Rating Scale
