# Supplementary material for: Study protocol for a randomised placebo-controlled trial of pramipexole in addition to mood stabilisers for patients with treatment resistant bipolar depression (the PAX-BD study)
Source: BMC Psychiatry. 2021 Jul 5;21:334. doi: 10.1186/s12888-021-03322-y (PMC8256234; doi:10.1186/s12888-021-03322-y)
Supplement: Supplementary file 1 — Additional file 1. [file 12888_2021_3322_MOESM1_ESM.pdf]

Appendix 1 – PAX-BD Patient Invite Letter V2.0 11/09/2020

[Site to send on letter headed paper including contact details]

Dear Patient

There is a research study taking place involving patients with Bipolar Disorder and I am contacting you to ask if you would be interested in receiving further information about this study?

I enclose a short leaflet which provides some more information about the research study. Not everyone who has Bipolar Disorder will meet the criteria to take part in the study but we would like to give you the chance to read this information so that you can decide whether you would like to find out more about it.

If you would like to discuss any part of the research with me then we can do so at our next appointment, or please get in touch with me on the number above. Alternatively you can contact the study team directly to discuss it in further detail.

Please complete and return the reply slip enclosed if you would like to receive further information about the study. This does not mean you are obligated to take part in the research study. All research is voluntary, you can always choose whether or not you want to be involved.

Once you have received the participant information sheet a member of the local study team would then be in touch to answer any questions.

Yours sincerely

Dr [XXXX]

……

Reply Slip:

☐ Yes I am interested in finding out more about the PAX-BD Study. Please send me a participant information Sheet.

Name:

Address:

Telephone No:

Post to:

A member of the local study team will telephone you to check you have received the information sheet and answer any questions.
