# Supplementary material for: Study protocol for a randomised placebo-controlled trial of pramipexole in addition to mood stabilisers for patients with treatment resistant bipolar depression (the PAX-BD study)
Source: BMC Psychiatry. 2021 Jul 5;21:334. doi: 10.1186/s12888-021-03322-y (PMC8256234; doi:10.1186/s12888-021-03322-y)
Supplement: Supplementary file 2 — Additional file 2. [file 12888_2021_3322_MOESM2_ESM.pdf]

Appendix 2 – PAX-BD Participant Summary Leaflet V1.0 23/07/2020

What does taking part in the study involve?

You will only enter the study if both you, and your mental health team as well as the study team agree it is right for you. Throughout the study, you will be in regular contact with the study team as well as with your usual clinical team. Taking part will not alter/affect any of your usual care.

Stage 1:

You will start with a review of your existing treatment and some changes to your medication will be made if necessary. You will have phone calls with a Research Assistant who will explain everything to you, including how to use the online system to record how you are feeling.

Stage 2:

You will be randomised to receive either placebo tablets "dummy tablets" or pramipexole tablets. This is a random decision, like a coin toss. Neither you, nor your own clinical team, and not even the study team will know which you are taking. This information *would* be available if needed. You will continue to receive the telephone calls and use the online system to monitor your wellbeing.

End of the Study:

You will take the study medication for up to 52 weeks, *as long as you and your doctor agree it is right to continue*. At the end of the study you will be able to discuss your treatment options with your clinical care team, including whether or not you want to continue to take pramipexole after the study ends (if that is what you have been taking)

If you are interested in taking part in the study please get in touch with your psychiatrist or Clinical Team and ask them to refer you to your local PAX-BD study team.

Local Study Team Details:

Principal Investigator:

TBC by site

CSO/Research Nurse:

TBC by site

The Newcastle Clinical Trials Unit, part of Newcastle University, are managing the study on behalf of the NHS sponsor, Cumbria, Northumberland, Tyne & Wear NHS Foundation Trust

Chief Investigator:
Prof Hamish McAllister-Williams

PAX BD
Pramipexole Trial for Bipolar Depression

A randomised, double-blind, placebo controlled trial of pramipexole in addition to mood stabilisers for patients with treatment resistant bipolar depression

NHS
Cumbria, Northumberland, Tyne and Wear
NHS Foundation Trust

Newcastle University

What is Pramipexole?

Pramipexole is a medication usually used to treat Parkinson's Disease or Restless Leg Syndrome. Previous research has suggested it might also be useful for treating depression.

The PAX-BD trial aims to discover if pramipexole is an effective treatment for bipolar depression in patients who have not improved with standard treatments.

The PAX-BD trial is a large study aiming to recruit 290 participants from across the UK. Participants will be randomly assigned either pramipexole or a placebo and then take the study medication for up to 48 weeks. It will not be known either way until the end of the trial.

Prior to starting the study medication participants need to start mood stabilising medication and stop any anti-psychotic medication.

Can I Take Part?

This study is for people with bipolar disorder who are currently depressed and for whom standard treatments have not yet worked. Can you answer yes to the following?

- ✓ A diagnosis of Bipolar Disorder (type I or II)
- ✓ You are currently experiencing a Major Depressive Episode
- ✓ You have failed to have responded to adequate trials of two NICE recommended medications (quetiapine, olanzapine (with or without fluoxetine), lamotrigine or lurasidone)
- ✓ You are aged 18 or older
- ✓ You are currently under the care of secondary care mental health services.
- ✓ You and your clinician think a change in medication might be helpful

Is taking part right for me?

The study team will check thoroughly if there are any reasons why you should not take part in the trial. You will only go ahead if they all feel it is safe for you to do so.

It might not be suitable for you to take part in the study if you have a serious health condition either which might be made worse by taking pramipexole.

You must not be pregnant nor planning to get pregnant, nor breast feeding during the trial.

You must not be taking part in another clinical trial at the same time, or about to start psychotherapy, as this might confuse the results of the trial.

Thank you for your interest.
