# Supplementary material for: Study protocol for a randomised placebo-controlled trial of pramipexole in addition to mood stabilisers for patients with treatment resistant bipolar depression (the PAX-BD study)
Source: BMC Psychiatry. 2021 Jul 5;21:334. doi: 10.1186/s12888-021-03322-y (PMC8256234; doi:10.1186/s12888-021-03322-y)
Supplement: Supplementary file 3 — Additional file 3. [file 12888_2021_3322_MOESM3_ESM.pdf]

Newcastle University

PAX BD
Pramipexole Trial for Bipolar Depression

Trust headed paper

A randomised, double-blind, placebo controlled trial of pramipexole in addition to mood stabilisers for patients with treatment resistant bipolar depression

The PAX-BD Study

INVITATION

We are inviting you to take part in a research study called the PAX-BD study. This information sheet tells you more about why it is being done and what it might mean for you. Please read the following information carefully to help you decide if you want to take part. You don't have to decide straight away and you can talk to your friends and family. Ask us if you have any questions or you want to know more.

KEY POINTS

- The study will look at the use of a drug called pramipexole for treatment of people with bipolar disorder who are currently depressed.
- In **stage 1** your doctor will help you stop taking any antipsychotic medication that you are already on, and start taking mood stabiliser(s), if you do not already.
- In **stage 2** you will be randomised to either the **placebo group** (a 'dummy' drug) or the **treatment group** (pramipexole). You will have an equal chance of being in each group.
- Your study doctor and the study team will not know which group you are part of or which drug you are taking until the end of the study.
- You will be asked to complete online questionnaires about your mood and behaviours throughout the study.
- You will have regular phone calls with the study research assistants, who will ask about your mood, behaviours, symptoms and medication.
- You will be asked to complete a participant diary to help track your medication use.
- If, at the end of the study, you find out that you have been taking pramipexole and this has helped with your bipolar depression, you may be able to continue to take it outside of the study by speaking with your doctor.
- Your doctor and study team will follow all local and national guidelines relating to Coronavirus and your safety.

Please read the following information for further details about the study if you are interested in taking part

The PAX-BD Study
PARTICIPANT INFORMATION SHEET

Why is PAX-BD needed?

Bipolar disorder (BD) occurs in about 2.5% of people, over a lifetime. There are currently limited treatment options available for patients with depressive symptoms. BD does not always respond to these treatments, and it can get even worse. Some of these drugs also have side effects such as sedation (feeling very sleepy) and gaining weight.

Pramipexole is a drug that is already approved for use in the NHS as a treatment for patients with Parkinson's disease or Restless Legs Syndrome. The safety and side effects of pramipexole are well known. It has some of the same effects as a chemical called dopamine, which occurs naturally in your body. Results from some research studies have shown that pramipexole improved the symptoms of depression in patients with Parkinson's, and in patients with BD when taken with mood stabilisers. However, these studies have been done with small numbers of patients and for short periods of time.

We want to find out if pramipexole is a beneficial treatment to patients with BD who have depression that has not responded to other treatments.

To do this we are looking for around 290 patients with BD who are currently depressed, from up to 40 different mental health Trusts across the UK to take part.

Why have I been invited to take part in PAX-BD?

You have been diagnosed with bipolar disorder, and previous recommended treatments for depression have not worked for you. You are aged 18 or over and your clinical team think that you could meet the criteria to take part.

Do I have to take part?

No, it is up to you to decide if you want to take part. If you choose not to, you will continue to get the standard treatment arranged by your doctor.

If you agree to take part, you can change your mind and withdraw from the study at any time without having to give a reason. It is important that you never suddenly stop taking your study medication. The team at your hospital will help you do this safely over several weeks.

Given the current Coronavirus pandemic, is it safe for me to take part?
We can reassure you that the study will follow all local and national guidelines for Coronavirus. Most of this study can be done from your own home using telephone or video to speak with the research team. You will only need to go into hospital twice during the whole study, unless there is a clinical or safety reason for you to do so. Your local team will be in touch with you before arranging any face to face visits which will then follow the most up to date guidance on social distancing. If you have any questions, at any time, about this study and Coronavirus please speak to your local team. Their contact details are listed at the end of this document.

What does taking part involve?

There are 2 stages of the PAX-BD study. If you progress to stage 2, we will give you a more detailed information sheet at that time to tell you about the next steps.

Stage 1

Your doctor will help you to stop taking any antipsychotic medication that you are already on, and start taking mood stabiliser(s), if you do not already. The amount of time that stage 1 takes will vary for everyone, but it will take at least 4 weeks. See p5 for details about stage 1.

Stage 2

You will be randomised to either the placebo group (a 'dummy' drug) or the treatment group (pramipexole). You will have an equal chance of being in each group. You will increase your dose of study medication gradually until you reach your personal maximum dose. You will be prescribed study medication over 52 weeks. See p6 for details about stage 2.

Study Visits

You will see a study doctor at the start of both stages. For the rest of the study, you will continue to see your usual doctor or care team at appointments as you normally would. In some cases, your usual doctor will also be a study doctor, so this will be the same person you usually have appointments with.

Telephone Calls

For both stages you will have regular phone contact with one or two members of the central study team called Research Assistants (RAs), who work for Cumbria, Northumberland, Tyne and Wear NHS Foundation Trust, and are based in Newcastle. They are collecting data for the study and will be asking you some questions.

True Colours: Online Questionnaires

For both stages you will need to login to an online system called True Colours and complete questions at set times. It can be accessed using a computer, smartphone or tablet. The system will remind you when you need to complete these. The RAs in Newcastle will be able to support you with this as well. Paper versions can be made available if necessary.

Pregnancy

To take part in the study, women must not be pregnant, breast-feeding or be planning a pregnancy during the time of the study. To prevent pregnancy during the study, all women of child bearing potential have to use what is called a 'highly effective' method of contraception.

These methods include:

- combined hormonal contraception (oral, intravaginal, transdermal)
- progestogen only hormonal contraception (oral, injectable, implantable)
- intrauterine device (IUD)
- intrauterine hormone-releasing system (IUS)
- vasectomised partner
- bilateral tubal occlusion
- Practice true abstinence in line with preferred and usual lifestyle

If you do become pregnant during the course of the study, you must tell your doctor **immediately** so appropriate action can be discussed. If you become pregnant during stage 1 of the study, you will not be able to proceed to stage 2.

STAGE 1

Study Call
You will receive a phone/video call from the hospital or clinic that you would normally attend for treatment. A study doctor will talk you through the study and answer any questions you might have. They will also speak with you about the risks of becoming pregnant whilst taking part in the study. We do not know what effect the drug would have on an unborn child so it is important that you do not become pregnant and are not planning to become pregnant throughout the study. You will be asked to confirm what contraception you are using and will use for the rest of the study.

For some patients, taking pramipexole can cause problems like using alcohol or drugs in a harmful way, binge eating, increased gambling or sexual behaviours. Patients who have experienced these problems in the past should not take part in the study. If you think this may affect you in any way, please tell the study doctor at this visit.

Consent Visit
If you would like to take part in the study, you will be asked to come into the hospital or clinic for a short appointment to sign a consent form.

You will be given a copy of the consent form to keep and a unique study ID number which will be used instead of your name on study documents. Only the study team and your local care team, will know this number links to you.

If you are female of childbearing potential you will be asked to give a urine sample to make sure you are not currently pregnant. The study doctor will speak to you about the risks of becoming pregnant whilst taking part in the study. You will be asked to confirm what contraception you are using and will continue to use for the rest of the study. We do not know what effect the drug would have on an unborn child so it is important that you do not, and are not, planning to become pregnant during the study.

Screening Call
You will receive a phone/video call from the hospital or clinic that you would normally attend for treatment. The call will take around 30 minutes. You can ask any questions you may have.

A study doctor will look through your medical history and ask you some questions. The doctor will then do some checks to confirm that you can take part in the study. This will include asking you to complete a questionnaire called the Quick Inventory of Depressive Symptomatology – Self Report (QIDS-SR). This will take about 5 minutes to complete. They will also ask you some questions about your symptoms and any medication that you take.

The doctor will confirm either way whether you can take part in stage 1 of the study. If you do not meet the criteria to take part in the study, you will not be able to take part in the study, and will continue under the care of your usual care team outside of the study.

If you do meet the criteria to take part, with your permission, we will inform your GP and your local care team that you are taking part in the PAX-BD study. We will send you a copy of this letter. A copy of this Information Sheet will also be sent with the letter. It will also be noted in your hospital medical records so that staff in the hospital know you are taking part in the study.

Optional interviews: we would like to find out about your views and experiences of PAX-BD. We might not be able to contact everyone, but if we do, one of the RAs will call you to arrange a convenient date and time to do the interview by telephone. This is separate to the main study, so you do **not** have to agree to be interviewed. See p.13 for further information about these optional interviews.

Withdrawing Antipsychotics and Starting Mood Stabilisers

- If you are taking antipsychotic drugs (such as quetiapine, aripiprazole, olanzapine, lurasidone and risperidone), you will be advised how to safely stop taking these, under the care of your local clinical team. This has to be done because antipsychotic drugs can block some of the effect of the study drug, pramipexole. This process may take longer for some people than others, but this will always be done safely.
- You will start to take one or more mood stabilisers (these will include lithium, lamotrigine, valproate, carbamazepine), if you are not already taking one or more of these medications.
- You need to reach and stay at a stable dose of mood stabilisers, and be off antipsychotics, for at least 4 weeks before you can progress to the next stage of the study.

Prescriptions during this stage will be charged to you as they would normally.

Weekly RA Telephone Calls and Questionnaires

After your clinic visit, one of the study Research Assistants (RAs) will call you within a couple of days to introduce themselves. They will check you have been able to login to the online system True Colours, or arrange for paper versions to be provided if required.

An RA will call you each week during stage 1. These calls will include asking you how you are getting on with the withdrawal of your antipsychotics and starting mood stabilisers (if applicable), guide you through any missing data/forms on True Colours, ask you what medication you are taking and if you have experienced any symptoms or site effects since starting in Stage 1 of the study.

Please note that the study RAs are not medically qualified and can’t advise you on your medication or on any symptoms. You should discuss any concerns with your doctor.

When you register with True Colours, you will be asked to fill in some initial information including your date of birth, gender, height and weight and will need to provide an email address. During stage 1, you will also need to complete weekly online questionnaires in True Colours about your depressive and mania symptoms and how these impact on your health. These weekly questionnaires should take around 10 to 15 minutes to complete each week. It is important that you speak with the RAs and complete your online questionnaires. This will help to make sure we can get results from the study and the data is accurate.

You will take part in stage 1 for a minimum of 4 weeks. The RAs will let your local study team know when they think you meet the criteria to be assessed for stage 2 of the study, and a screening and randomisation visit will be arranged at your local clinic or at your home. You will continue to get weekly calls until this visit takes place and **you must still continue to complete your online questionnaires.**

You will be given a **Randomisation Information Sheet** before this visit that will give you even more detailed information on the next steps.

STAGE 2

Study Call The study doctor will call you at home to go through the Randomisation Information Sheet with you and answer any questions you might have.

Consent Visit

If you want to continue in the study the study doctor will ask you to come to the hospital or clinic for a short appointment where they will ask you to sign another consent form and you will be given a copy to keep.

If you are female of childbearing potential you will again be asked to provide a urine sample to confirm that you are not currently pregnant.

Screening Call

You will receive a phone/video call from your hospital or clinic that you would normally attend for treatment The study doctor will do some checks to confirm if you continue to take part in the study. This will include asking you to complete a questionnaire called the Quick Inventory of Depressive Symptomatology – Self Report (QIDS-SR). This will take approximately 5 minutes to complete.

There may be other tests that your doctor thinks are needed before they can confirm if you can go to the next stage, such as a blood test, but this will be the decision of your study doctor. The doctor will let you know either way if you are able to continue. If the doctor is happy that you meet the criteria for the study they will confirm you as what we call 'eligible'. Being eligible means you can then complete your first assessments for stage 2 and a member of the team will randomise you to one of the groups.

If you are not eligible at this point, you will not be able to take part in the study, and will continue under the care of your usual care team outside of the study. Thank you for your interest in the study.

Baseline Assessments

Baseline means these are your first assessments and are taken before you start taking your study medication.

Following your visit, a RA will call you to talk to you further about what will happen next, explain when you will be getting your study medication, and you will need to complete some extra online questionnaires at this time.

Randomisation

Group 1: Placebo

If you are put in group 1, you will be given a placebo. This is a 'dummy' drug. It looks exactly the same as the real drug but it is made with non-active ingredients.

Group 2: Pramipexole

If you are put in group 2, you will be given pramipexole. Only patients in this group will receive pramipexole.

- You will have an equal chance of being in group 1 or group 2 (a 50:50 chance). Your group will be picked by a computer. We call this randomisation. Your doctor and treating team will not have any say on which group you are put in.

- To make it a fair comparison, you won't know which group you are in, neither will your doctor, the RAs or the PAX-BD team. This will stay unknown until close to the end of the study, unless there is a clinical reason or emergency that means this information is needed for your safety.

Study Medication (Placebo or Pramipexole)

When we talk about study medication, this includes the placebo as well as the pramipexole. You will be prescribed doses of study medication by the clinical team over a maximum time of 52 weeks. This will be posted to you from the Cumbria, Northumberland, Tyne and Wear NHS Foundation Trust pharmacy by Royal Mail and you will need to sign for the delivery. The prescription of this study medication is free of charge for you, but any other prescription (including mood stabilisers) will be arranged and charged to you as they would normally.

Medication Schedule

- **Week 1** of stage 2 starts when you begin to take the study medication.
- In **week 1** you will start on a low dose. This will be increased over the first 4 weeks to a maximum dose. You will be given instructions on how to increase your dose.
- Once you have reached your personal maximum dose, you will stay on this from **week 5** to **week 12**, unless there is a clinical reason this needs to change.
- After **week 12**, your dose may change and this will be discussed and decided by you and your clinical team.
- At **week 46**, you will be asked to think about if you want to stay on study medication after the study has ended. You can talk about this with your clinical team.
- At **week 48**, you will not automatically find out if you have been taking the active study drug pramipexole, or the dummy placebo drug during the study. However if you would like to continue taking pramipexole outside of the study, the local team can make arrangements to find out what you have been taking. In this case:
    - If you have been taking **placebo**, you will not need to continue taking it and can stop when your clinical team tells you to do so.
    - If you have been receiving placebo but would like to try taking pramipexole outside of the trial you will be able to discuss this option with your clinician as part of your ongoing clinical care.
    - If you have been taking **pramipexole** and you have said you **do** want to continue taking it after the end of the study, your clinical team will have time to try to make arrangements for this medication available to you outside of the study. Enough study medication will be prescribed until the end of **week 52**.
    - If you have been taking pramipexole and then decide you do **not** want to continue taking it after the end of the study, your clinical team will discuss with you how to safety reduce your medication over several weeks before you can stop.
    - If at week 48 you do not want to continue taking pramipexole outside of the study, the team will not find out for you at this stage what you have been taking during the study. Arrangements can be made for you to find out once the study has completely finished if you would like to know this.

Never stop your study medication suddenly. Always speak to your study doctor or local clinical team first about how to do stop safely. They will help you to safely reduce and stop the medication over several weeks and they will monitor your health as you do this.

Participant Diary

You will be given a printed diary to complete. You will write in here the dose of study medication you have taken, other medications you may be taking, and any symptoms or medical events you have had (anything from headaches to breaking a bone). The RAs will ask you for this information when they call you, so the diary will help you keep track.

Weekly RA Telephone Calls and Questionnaires

During stage 2, you will need to continue to complete weekly questionnaires and speaking to the RA on the phone when they call you (weekly for the first 12 weeks of stage 2, and then every 4 weeks until week 48, and then weekly until you stop taking study medication).

Collecting empty / left over bottles of medication

You will be asked to return any empty or leftover bottles of medication seven times during the 52 weeks of the study. This can be done either by dropping them off at your local hospital / clinic, arranging collection with your clinical team or by sending them through the mail.

When the final empty or leftover bottles of medication are returned, if you are a female of childbearing potential, you will again be asked for a urine sample to confirm that you are not currently pregnant.

The study team will report to your clinical team if these results raise any concerns. You will continue to see your doctor for clinic appointments as usual.

Side Effects

As with any medicine, the medication used in this study may cause side effects.

**Some very common side effects of pramipexole that have been reported are:**
• Drowsiness/sleepiness<br>• Dizziness<br>• Abnormal or uncontrolled involuntary movements<br>• Nausea
**<u>Other commonly</u> reported side effects of pramipexole include:**
• Trouble sleeping (insomnia), hallucinations, abnormal dreams, confusion, impulse control behavioural symptoms<br>• Headache<br>• Visual impairment such as double or blurred vision, or reduced clarity<br>• Low blood pressure<br>• Constipation<br>• Vomiting<br>• Fatigue (tiredness)<br>• Fluid accumulation (oedema)<br>• Weight loss<br>• Decreased appetite
**<u>Other uncommon</u> <u>or rarely reported</u> side effects of pramipexole include:**
• Dopamine agonist withdrawal syndrome: this includes a lack of interest/enthusiasm, anxiety, depression, fatigue, sweating and pain<br>• Pneumonia<br>• Psychiatric symptoms such as compulsive shopping, pathological gambling, restlessness, hyper sexuality, delusion, libido disorder (increased or decreased), paranoia, delirium, binge or excessive eating, mania<br>• Sudden onset of sleep<br>• Memory loss (amnesia)<br>• Muscle spasms<br>• Fainting<br>• Heart failure<br>• Difficulty breathing<br>• Hiccups<br>• Skin hypersensitivity (rash/itch)

If you experience any of these side effects it is important that you let your doctor know straight away. The study doctor can talk to you more about what these mean if you are unsure about any of them. The side effects experienced may only be temporary as you adjust your dose but it is important to discuss these so that they can be monitored.

Driving

Taking pramipexole can have a major influence on the ability to drive and use machines. It can make you feel drowsy or sleepy and have episodes of suddenly falling asleep. If you experience this at any time whilst you are taking the study medication, you **must not** drive or take part in any thing where not being alert might put you at risk of serious injury or death (like operating machines). You should tell your doctor if this occurs.

Further Supporting Information

Expenses and payments

As a thank you for taking part in the study, you will be provided with gift vouchers of £25 by post at weeks 12, 36 and 52.

What happens when the research study stops?

At the end of the study you will continue with your standard clinical care. This may include taking pramipexole if this has been arranged by your local clinician.

We hope that the results of this study will help us say if pramipexole is beneficial for patients with BD. You will be able to find out about the study results from the Northern Centre for Mood Disorders' website and through Bipolar UK. We will send you a copy of study results if you agree to this.

What are the benefits of taking part?

We cannot promise the study will help you directly but the information we get from this study may help to improve the treatment for people with treatment resistant bipolar depression. If you want to find out more about taking part in research studies you can visit the NHS Choices website www.nhs.uk.

What are the possible disadvantages or risks of taking part?

We want you to be safe in this study at all times, but all medical treatments carry some risk. Pramipexole is used by thousands of NHS patients with Parkinson's Disease or Restless Legs Syndrome, and there are some known side effects.

If you react badly to the drug your doctor will be able to change your medication and treat you to try to alleviate your symptoms. If they need to find out which treatment you are taking (pramipexole or placebo), this information is available in case of an emergency.

What will happen if I don't want to carry on with the study?

You can withdraw from the study medication and carry on completing the online questionnaires, or withdraw from the study completely. You can withdraw at any time for any reason, without giving a reason. You will be fully cared for and supported in line with your hospital's standard practice.

We ask if you are happy for us to:

- continue collecting information about you until the end of the study

- record why you decided to withdraw.

If you withdraw from the study, we will keep the information about you that we have already obtained.

You must never suddenly stop taking the study medication.

What if there is a problem?

If you have a concern about any aspect of this study, you can speak to a member of the study team (this could be at your hospital or clinic, or one of the RAs) who will do their best to answer your questions. Further contact details are included at the end of this information sheet. If you are still unhappy and want to raise your concerns with someone who is not directly involved in your care, you can contact <site to localise with local details such as PALS phone number and email address>

In the unlikely event that you are harmed during the study and this is due to someone's negligence (they were careless) you may have grounds for legal action and compensation, but you may need to meet your own legal costs. NHS Indemnity does not offer no-fault compensation (for harm that is not anyone's fault).

The Newcastle Clinical Trials Unit, part of Newcastle University, are managing the study on behalf of the study NHS sponsor. Newcastle University also have insurance arrangements in place to cover Newcastle University staff involved in designing and managing the PAX-BD study.

What will happen to the results of the research study?

- The results will be published in medical journals and presented in meetings to other doctors, nurses, researchers and patients.
- A report will be written for the study funder and put on their website.
- All study data that is published will be anonymous. Your identity will always be protected.
- The results will be available at the end of the study through publications, in the wider press and directly to patient BD groups e.g. via Bipolar UK.
- Fully anonymised data may be made available to other researchers both within and outside the UK to help inform other research studies.

Will my taking part in this study be kept confidential?

Yes. All of the information collected will be entered on computers that are kept secure and password protected.

- You will be given a unique identification number instead of writing your name on study documents. Staff at your hospital/clinic will be able to link this number back to you using your date of birth, name and NHS number.
- Information with your ID number will only be shared with researchers on the study team, both in and outside of the UK.
- The Sponsor, Cumbria, Northumberland, Tyne and Wear NHS Foundation Trust (CNTW):
    - We will use your contact details for the RA telephone calls
    - Pharmacy will have access to your contact details for posting your study medication
- Your local study doctor will be contacting you about your study medication and your health.

- Your email address will be used for sending the True Colours reminders.
- Your name and address will be provided to Royal Mail so that your study medication can be posted to you
- Your contact details will never be shared with anyone else outside of the study.
- You will not be named in any results, reports or anything on our website.
- Very occasionally, information might be given during the study that we would have a legal obligation to pass on to others (for instance information which suggested you or others were at risk of harm). In this case, confidentiality would be broken so that we could pass this information to the relevant people. You would be informed of this.
- Site staff will have access to your information during the study to organise trial visits as well as for ongoing safety. At the end of the study, all trial information will be kept in a secure storage area (this is called archiving) for at least 5 years. This makes sure any queries about the running of the study have been answered. All information will be held securely to make sure we protect your confidentiality, after which it will be safely destroyed.
- We will ask your permission for a copy of the completed consent form to be sent securely to the Newcastle Clinical Trials Unit (NCTU). This is so that the NCTU team can carry out planned checks of completed forms. This is optional and you can write on the consent form if you agree to this or not.

Who is organising and funding the research?

The central study doctor (also known as the 'Chief Investigator') is Professor Hamish McAllister-Williams, a Consultant Psychiatrist and Professor of Affective Disorders. He is based in Newcastle upon Tyne. The study team also include senior doctors and nurses, university experts in research studies and members of the public.

Study sponsor: Cumbria, Northumberland, Tyne and Wear NHS Foundation Trust. PAX-BD is managed by the Newcastle Clinical Trials Unit on behalf of the sponsor.

Study funder: the National Institute for Health Research (NIHR) Health Technology Assessment (HTA) programme (reference 16/154/01). This body is funded by the UK government to carry out research for the benefit of the NHS and its patients.

Up to 40 trusts will be taking part in the PAX-BD study. Each trust will have a study doctor, called an Investigator. The investigator for your trust is ____________________

Who has reviewed the study?

The funder reviewed the study plan as part of the application for funding. All research in the NHS is also looked at by an independent group of people, called a Research Ethics Committee, to protect your interests. This study has been reviewed and given favourable opinion by North East - Newcastle & North Tyneside 2 Research Ethics Committee.

Patients and carers, including from a Bipolar Support Group, have also been involved in deciding how to do PAX-BD. We also asked a group of patients with BD to look at the information sheets to make sure they are presented in a clear way, are easy to understand, and include important information. We also have patients on the different study committees who help oversee the running of the study.

Who is providing the study drug?

The study sponsor pharmacy will be sending the study medication (pramipexole or placebo) to PAX-BD participants in the post. They are based in Newcastle upon Tyne. A company called ModePharma has provided the study drug and made the placebo to match for this study.

What if relevant new information becomes available?

If, during the course of the study, new information becomes available that is relevant to you, we will tell you about it and discuss whether you should or would like to withdraw from the study. If it is better for you to withdraw, you can do this without giving a reason. This will not affect the care that you receive.

Thank you for taking the time to read this information sheet, and for your interest in the PAX-BD study. Please see the team contact details below and a study diagram on the next page.

http://www.mood-disorders.co.uk/PAX-BD

[LOCAL CONTACT DETAILS]

Further Information about the Optional Telephone Interviews

We would like to hear about peoples' experiences of taking part in PAX-BD. We want to speak to as many people as we can, but we will not be able to speak to everyone involved in the study. This is separate to the main study, so you do **not** have to agree to be interviewed. Please indicate on the study consent form whether or not you would like to be contacted for an interview. If we do contact you, a RA will get in touch to arrange a convenient date and time to do the interview.

- The interview will be over the telephone.
- The interview will last about 30 minutes, no longer than an hour (depending on how much you have to say). It will be conducted by a RA.
- We will ask your permission to audio-record the conversation so that the interviewer can talk to you without having to make too many notes.

How will the interview be used?

The conversation will be written out in full (transcribed) and used by the researchers to improve this and future studies. Your name and any personal details will be removed from the written version to make sure you cannot be identified (anonymised). We hope to publish the results of the study in scientific journals, which may include anonymised quotes from the conversations.

Will what I tell you be kept confidential?

- Any recordings of your voice and transcripts (written out version) will be kept securely on a secure password protected database at Cumbria, Northumberland, Tyne and Wear NHS Foundation Trust and University of Nottingham.
- The recordings will be shared with a professional transcription company to be written out in full.
- All recordings will be deleted at the end of the study.
- We will use a number to identify you instead of your name (we call this pseudo-anonymised). We will remove anything else that could identify you (anonymised).
- The anonymised transcripts will be kept to help with future research. We may want to use quotes from the transcripts but they will not include your name.
- Information about you will be looked at by people directly involved in the study, as well as by people who are checking it is running as it should.

Stage 1

Phone/video call with study doctor
- Discussion about the study, contraception & any questions you might have

Visit with study doctor
- Written consent to stage 1 (including consent to optional interviews)

Urine pregnancy test (if applicable)

Phone / video call with study doctor
- Questions about your medical history & completion of questionnaires
- Study doctor confirms if you are suitable to take part

STAGE 1
- Anti-psychotics stopped & mood stabilising medication started (if applicable)
- Weekly telephone calls with Research Assistants
- Registration to True Colours and completion of weekly questionnaires
- Stage 1 will last for a minimum of 4 weeks

Stage 2

Phone/video call with study doctor
- Discussion about stage 2, contraception & any questions you might have

Visit with study doctor
- Written consent to stage 2

Urine pregnancy test (if applicable)

Phone / video call with study doctor
- Completion of questionnaires and study doctor confirms you can continue

STAGE 2
- Week 1 starts when you start taking study medication
- Study medication taken for up to 52 weeks
- Medication sent by (signed for) post to your chosen address

Study medication
- Allocated at random to group 1 or group 2 (but won't know which)
- Weeks 1-4: Dose increased gradually until personal maximum dose is reached
- Weeks 5-12: Stay on personal maximum daily dose
- Weeks 13-48: Flexible dose as decided by doctor in discussion with you

Group 1
Placebo (dummy drug) + Mood stabilisers

Group 2
Pramipexole + Mood stabilisers

Telephone calls
Weeks 1-12: weekly; Weeks 13-48: every 4 weeks;

Weeks 48-52: weekly until study medication is stopped

CSO contacts (weeks 2, 6, 12, 24, 36, 48, 52)
Collection of unused medication and empty bottles

Week 52 only – pregnancy test (if applicable)

Online Questionnaires
Weekly completion using True Colours system

Extra questionnaires to complete at some weeks

End of stage 2 (week 48 -52)
- Discussion with study doctor on whether you would wish to continue to take pramipexole outside of the study
- Participants who do wish to continue taking pramipexole outside of the study can find out if they were receiving placebo or pramipexole
- Pregnancy test (if applicable)

End of study
- Continue to receive standard care provided by your usual clinical team
- This may include taking pramipexole outside of the study if approved locally

Data Protection and Transparency Information

Cumbria, Northumberland, Tyne and Wear NHS Foundation Trust (CNTW) is the sponsor for this study based in the United Kingdom and will act as the “data controller” for this study. **They are responsible for looking after your information and using it properly.**

This study is managed on behalf of the sponsor by the Newcastle Clinical Trials Unit who will act as the “data processor”. As data processor, this means that we are responsible for processing personal data on behalf of a controller. We will be using information from you in order to undertake this study, and will keep identifiable information about you for 5 years after the study has finished.

TrueColours will act as a data processor for the study and the information that you enter on to the online platform will be stored securely on servers of the University of Oxford. This information will be transferred securely to the Sponsor and study researchers who will analyse your data together with the data of all the other participants. This data will be provided to the researchers in a pseudo-anonymised form, will be held securely and kept confidential. The information that you provide will be held for 5 years after the study has finished.
Royal Mail will act as a data controller for this study. The sponsor will provide Royal Mail with your name and the address you have provided to allow Royal Mail to deliver your study medication to you. Royal Mail has internal data retention policies which cover secure destruction of all information in compliance with their legal and regulatory obligations. Data provided to Royal Mail will be stored for a maximum of 13 months and 1 day before being destroyed. Royal Mail Group’s policy is to only retain information for as long as it is required for the purpose or purposes for which they use it.
Your rights to access, change or move your information are limited, as we need to manage your information in specific ways in order for the research to be reliable and accurate. If you withdraw from the study, we will keep the information about you that we have already obtained. To safeguard your rights, we will use the least amount of personally-identifiable information possible.

You can find out more about how your information is used at
www.cntw.nhs.uk/about/research/

To find out more about research and general use of patient information please refer to the Health Research Authority Website https://www.hra.nhs.uk/information-about-patients/

The local study team at your hospital or clinic and the central Research Assistants (RAs) will collect information from you and/or your medical records for this research study in accordance with our instructions.

The local study team and RAs will use your name, NHS number and contacts details to contact you about the research study, and make sure that relevant information about the study is recorded for your care, and to oversee the quality of the study. Individuals from the sponsor, Newcastle Clinical Trials Unit and regulatory organisations may look at your medical and research records to check the accuracy of the research study. The local study team will pass these details to the sponsor or the Newcastle Clinical Trials Unit along with information collected from you and/or your medical records. The only people at sponsor or the Newcastle Clinical Trials Unit who will have access to information that identifies you will be people who

need to contact you or audit the data collection process. The people who analyse the information will not be able to identify you and will not be able to find out your name, NHS number or contact details.

When you agree to take part in a research study, the information about your health and care may be provided to researchers running other research studies in this organisation and in other organisations. These organisations may be universities, NHS organisations or companies involved in health and care research in this country or abroad. Your information will only be used by organisations and researchers to conduct research in accordance with the UK Policy Framework for Health and Social Care Research.

This information will not identify you and will not be combined with other information in a way that could identify you. The information will only be used for the purpose of health and care research, and cannot be used to contact you or to affect your care. It will not be used to make decisions about future services available to you, such as insurance.

If you join the study, identifiable information about you will be stored and processed at your local hospital/clinic, the sponsor's Trust and Newcastle Clinical Trials Unit. All information which is collected about you during the course of the research will be stored in secure and locked offices, on a password-protected database and on secure Trust and University servers.
