# Supplementary material for: Study protocol for a randomised placebo-controlled trial of pramipexole in addition to mood stabilisers for patients with treatment resistant bipolar depression (the PAX-BD study)
Source: BMC Psychiatry. 2021 Jul 5;21:334. doi: 10.1186/s12888-021-03322-y (PMC8256234; doi:10.1186/s12888-021-03322-y)
Supplement: Supplementary file 5 — Additional file 5. [file 12888_2021_3322_MOESM5_ESM.pdf]

Newcastle University

PAX BD Pramipexole Trial for Bipolar Depression

Trust headed paper

A randomised, double-blind, placebo controlled trial of pramipexole in addition to mood stabilisers for patients with treatment resistant bipolar depression

The PAX-BD Study – STAGE 2 KEY POINTS

- You have now **completed stage 1**, we hope you will be happy to progress to **stage 2**. **Stage 2 lasts for 1 year.** You will be allocated at random to take either **placebo medication** (a 'dummy' drug) or **pramipexole**. **See page 5.**

- Study medication will be sent to you by post (approx. every 8 weeks). You will be given clear instructions on how to gradually increase your dose to find the daily dose that you are happy with. You will continue to take your mood stabilising medication. **See page 6.**

- Women must not be pregnant, breast-feeding or be planning a pregnancy during the study. **See page 4 for a list of appropriate contraception (females only).**

- You will be asked to complete a diary to keep track of the medication you are taking. This shouldn't take you more than a minute or two each day. Your doctor can show you what the diary looks like.

- If you have missed a dose of study medication, but remember within 12 hours of the time you should have taken the dose, take that dose straightaway. If you remember more than 12 hours later, do not take anything until your next dose. Never take a double dose to make up for a forgotten tablet dose.

- You will continue to complete online weekly questionnaires about your mood and behaviours and receive phone calls from study Research Assistants (RAs) (weekly for 12 weeks, and then every 4 weeks).. **See page 2 for a diagram of visits.**

- At the end of the stage 2, if you want to continue taking pramipexole you may be able to continue to take it outside of the study by speaking with your doctor. **See page 11 for end of study information.**

- As with any medicine, the medication used in this study may cause side effects. **A list of possible side effects can be found on page 12.**

- **You must never suddenly stop taking the study medication.** If you decide at any time that you want to or are advised to stop taking study medication, your doctor will advise on how to gradually reduce the dose until you can safely stop taking it.

- Your doctor and study team will follow all local and national guidelines relating to Coronavirus and your safety.

If you have any queries about the PAX-BD study or your study medication, please contact your local study team:

LOCAL STUDY TEAM CONTACT DETAILS

NHS Cumbria, Northumberland, Tyne and Wear NHS Foundation Trust

The PAX-BD Study – STAGE 2 VISIT DIAGRAM

Stage 1

Phone/video call with study doctor
- Discussion about the study, contraception & any questions you might have

Visit with study doctor
- Written consent to stage 1 (including consent to optional interviews)

Urine pregnancy test (if applicable)

Phone / video call with study doctor
- Questions about your medical history & completion of questionnaires
- Study doctor confirms if you are suitable to take part

STAGE 1
- Anti-psychotics stopped & mood stabilising medication started (if applicable)
- Weekly telephone calls with Research Assistants
- Registration to True Colours and completion of weekly questionnaires
- Stage 1 will last for a minimum of 4 weeks

Stage 2

Phone/video call with study doctor
- Discussion about stage 2, contraception & any questions you might have

Visit with study doctor
- Written consent to stage 2

Urine pregnancy test (if applicable)

Phone / video call with study doctor
- Completion of questionnaires and study doctor confirms you can continue

STAGE 2
- Week 1 starts when you start taking study medication
- Study medication taken for up to 52 weeks
- Medication sent by (signed for) post to your chosen address

Study medication
- Allocated at random to group 1 or group 2 (but won't know which)
- Weeks 1-4: Dose increased gradually until personal maximum dose is reached
- Weeks 5-12: Stay on personal maximum daily dose
- Weeks 13-48: Flexible dose as decided by doctor in discussion with you

**Group 1**	**Group 2**
Placebo (dummy drug) + Mood stabilisers	Pramipexole + Mood stabilisers

**Telephone calls**	**CSO contacts** (weeks 2, 6, 12, 24, 36, 48, 52)	**Online Questionnaires**
Weeks 1-12: weekly; Weeks 13-48: every 4 weeks;<br><br>Weeks 48-52: weekly until study medication is stopped	Collection of unused medication and empty bottles<br><br>Week 52 only – pregnancy test (if applicable)	Weekly completion using True Colours system<br><br>Extra questionnaires to complete at some weeks

End of stage 2 (week 48 -52)
- Discussion with study doctor on whether you would wish to continue to take pramipexole outside of the study
- Participants who do wish to continue taking pramipexole outside of the study can find out if they were receiving placebo or pramipexole
- Pregnancy test (if applicable)

End of study
- Continue to receive standard care provided by your usual clinical team
- This may include taking pramipexole outside of the study if approved locally

PAXBD
Pramipexole Trial for Bipolar Depression

A randomised, double-blind, placebo controlled trial of pramipexole in addition to mood stabilisers for patients with treatment resistant bipolar depression

The PAX-BD Study
Randomisation Information Sheet

This information sheet will give you some **more detailed information** about what happens during **stage 2** of the PAX-BD study. It will tell you more about the study visits, assessments, when you will be sent your medication and what happens at the end of the study. Please consider the information to make sure you understand what taking part would mean for you.

It is important to know that you can change your mind and not go any further.

Feel free to talk to others about taking part. Please ask us if there is anything that is not clear to you. **If you decide to take part, you can keep this information sheet and use it as guidance during the study.**

Why have I been invited to continue to take part in the PAX-BD study?
You have taken part in stage 1 of the PAX-BD study for at least 4 weeks. As part of this initial stage of the study you have stopped taking any antipsychotic drugs (if applicable) and have been taking a stable dose of mood stabilising medication. You have also completed the required study questionnaires and Research Assistant (RA) phone calls.

Do I have to take part?
You do not have to take part in the stage 2 and it is up to you to decide. If you choose not to, you will continue to get the standard treatment arranged by your doctor.

If you agree to take part, you can change your mind and withdraw from the study at any time. **It is important that you never suddenly stop taking your study medication.** If you decide to withdraw from the study please let your clinical team know so that they can make sure you do this safely (see p6, 'Stopping medication early and withdrawing' for further details).

Given the current Coronavirus pandemic is it safe for me to take part?
We can reassure you that the study will follow all local and national guidelines for Coronavirus. Most of this study can be done from your own home using telephone or video calls to speak with the research team. You will only need to visit the hospital/clinic for a study visit twice, unless there is a clinical or safety reason. Your local team will be in touch with you before arranging any face to face visits. All visits will follow the most up to date guidance on social distancing. If you have any questions, at any time, about this study and Coronavirus please speak to your local team.

What will happen to me if I continue in the study?
You have completed the first stage of the study and the study doctor can check if you now meet the criteria for stage 2 of the study. The visits are described in more detail below.

Study call

Your study doctor will call you to discuss the next stage of the study. This phone call will last about 30 minutes and they will go through the Randomisation Information Sheet with you. You will be able to ask any questions that you have and make sure you understand what the study means for you.

Contraception discussion: to take part in the study, women must not be pregnant, breast-feeding or be planning a pregnancy during the time of the study. If you are a female of childbearing potential, the study doctor will talk to you about the risks of becoming pregnant during the study. This is because we don't know what effect the study drug would have on an unborn child. You will need to confirm what contraception you are using and will use for the rest of the study.

All women of child bearing potential have to use what is called a 'highly effective' method of contraception.

These methods include:

- combined hormonal contraception (oral, intravaginal, transdermal)
- progestogen only hormonal contraception (oral, injectable, implantable)
- intrauterine device (IUD)
- intrauterine hormone-releasing system (IUS)
- vasectomised partner
- bilateral tubal occlusion
- Practice true abstinence in line with preferred and usual lifestyle

Consent Visit

If you want to continue in the study the study doctor will ask you to come to the hospital or clinic for a short appointment where the doctor will ask you to sign a consent form and you will be given a copy to keep.

Pregnancy test: if you are a female of childbearing potential we will ask you for a urine sample to confirm that you are not currently pregnant.

Screening Call

You will receive a phone/videocall from your hospital or clinic that you would normally come to for treatment The study doctor will do some checks to confirm whether or not you are eligible to take part in the study.

Questionnaire: You will be asked to complete a short questionnaire called the Quick Inventory of Depressive Symptomatology – Self Report (QIDS-SR). This will take about 5 minutes to complete.

There may be other tests that your doctor thinks are needed before they can confirm if you can continue to the next stage, such as a blood test, but this will be the decision of your study doctor. The doctor will let you know either way if you are able to continue. If the doctor is happy that you meet the criteria for the study they will confirm you are eligible to complete your first assessments for stage 2. At this point a member of the team will randomise you to one of the groups.

If you don't meet the necessary criteria, you will no longer be able to take part in the study. You will continue as before under the care of your usual care team outside of the study.

Optional interviews: we would like to find out about your views and experiences of PAX-BD. We might not be able to contact everyone, but if we do, one of the RAs will call you to arrange a convenient date and time to do the interview. This is separate to the main study and is optional, so you do **not** have to agree to be interviewed. Please see page 17 for further information about optional interviews.

STAGE 2

During the study, your care with your usual clinical team or doctor will continue as it would normally, and you will continue with any usual clinic appointments.

Online Questionnaires: You will need to **continue completing the online questionnaires that you completed in stage 1,** as well as some **additional** questionnaires that will now be available to you in the system. You should answer these additional questionnaires **<u>before</u>** you start to take the study medication (see 'True Colours: Online Questionnaires' on p8 for more information about these questionnaires).

Patient Safety Card: You will be given a patient card that includes contact information for your study team that can be used in case of an emergency. It is very important that **you carry this with you at all times** and present it to medical staff in an emergency.

Study Diary: You will be given a study diary to complete every day when you start taking your study medication. It shouldn't take you long to fill in. It will help you to remember what you doses you have taken, any you have missed, if you are experiencing any side effects or any symptoms, other medications you are taking (for any reason) and any supplements or over-the-counter medication you may be taking.

Study Groups: Once it has been confirmed that you are eligible to take part in stage 2, you will be allocated at random to Group 1 or Group 2.

Group 1: Placebo
If you are put in group 1, you will be given a placebo. This is a 'dummy' drug. It looks exactly the same as the real drug but it is made with non-active ingredients.

Group 2: Pramipexole
If you are put in group 2, you will be given pramipexole. Only patients in this group will receive pramipexole.

You will have an equal chance of being in group 1 or group 2 (a 50:50 chance). Your group will be picked by a computer. We call this 'randomisation'. Your doctor and treating team will not have any say on which group you are put in.

- To make it a fair comparison, you won't know which group you are in, neither will your doctor, the RAs or the PAX-BD team. This will stay unknown until close to the end of the study, or unless there is a clinical reason or emergency that means this information is needed for your safety.

- You will continue to take mood stabilising medication for the duration of the study, unless your doctor decides that a change in medication is required.

An RA will call you to confirm your allocation group, explain what happens next and when you will be getting your study medication, and ask you some questionnaires over the phone (see p7 for further information about phone calls).

Study Medication

When we talk about study medication, this includes the placebo as well as the pramipexole. These have been designed to look the same so that you can't tell which group you have been allocated to. You will be prescribed study medication by your study doctor over a maximum time of 52 weeks. This will be posted to you from the Cumbria, Northumberland, Tyne and Wear NHS Foundation Trust pharmacy by Royal Mail and you will need to sign for the delivery. The prescription of this study medication is free of charge for you, but any other prescription (including mood stabilisers) will be arranged and charged to you as they would normally.

You should keep your medication in a safe place and out of the reach of children.

Week 1 of stage 2 starts when you begin to take the study medication. There will be **7** lots of study medication sent to you during the study. Your study medication will usually arrive around a week before it is needed, see the table below:

Study Medication will be posted at these times:	To be taken in these weeks:
Before week 1	Weeks 1-5 (5 weeks)
By the start of week 5	Weeks 6-12 (7 weeks)
By the start of week 12	Weeks 13-20 (8 weeks)
By the start of week 20	Weeks 21-28 (8 weeks)
By the start of week 28	Weeks 29-36 (8 weeks)
By the start of week 36	Weeks 37-44 (8 weeks)
By the start of week 44	Weeks 45-52 (8 weeks)

A study RA will let you know when your medication is on its way. It is important that you don't stop taking medication even just for a few days, so please let the RA or your doctor know if your next batch of medication has not arrived when you are expecting it.

You should start taking the new medication at the start of the weeks listed above, even if there are some tablets left in the old bottles. Please don't start taking the new batch of study medication early as you may run out of tablets. Your study diary contains reminders to when you should start taking new bottles of medication.

As part of the research study, all bottles of medication need to be returned, whether they are empty or if they have tablets left. A returns envelope will be included with each delivery of medication. When you start taking a new lot of medication, please place old bottles in the envelope (whether they are empty or they have tablets left in them) and return in the mail, or hand over when you next see a member of the study team.

It is possible that you may be sent further prescriptions in addition to this, for example if you change dose during the study and don't have enough tablets. The study doctor will make sure you have enough tablets when discussing your change of dose.

What will the medication look like?

There are two sizes of tablet and each size will come in a different bottle. The bottle labels will be different colours to help you tell them apart. All of the tablets are white and flat and you should be able to swallow them easily, but are slightly different shapes depending on their size.

1mg (larger tablet) blue label –round shaped
0.25mg (smaller tablet) yellow label – oval shaped

Each bottle will have 56 tablets.

Medication Schedule

Weeks 1 to 4

- Your dose will start at the lowest level of 0.25mg daily.
- It will increase by 0.25mg every 3 days.
- This will continue until you reach a maximum of 2.5mg, or the highest dose that you can safely tolerate if this is lower.

We will give you a **dose schedule** with your medication. This tells you many of each size tablet you need to take each day to make your correct dose. We have also included a handy table in your study diary. It's really important you follow these instructions only to make up your daily dose. There may not be enough tablets if you use other combinations.

Weeks 5 to 12

You will continue to take the same daily dose you were taking at the end of week 4 (unless otherwise advised by your study doctor).

Weeks 13 to 48

Your study doctor will review how you respond to and tolerate the study medication. They will talk to you about whether to increase or decrease your daily dose (it will not go any higher than 2.5mg daily). You can discuss this with the clinical team and make a decision together about what is the best dose for you to continue taking.

Weeks 48 to 52

You will not automatically find out if you have been taking the active study drug pramipexole, or the dummy placebo drug during the study. However if you would like to continue taking pramipexole outside of the study the local team can make arrangements to find out what you have been taking.

In this case if you have been taking the placebo, you can stop taking it when your clinical team tells you to do so. If you have been receiving placebo but would like to try taking pramipexole outside of the trial you will be able to discuss this option with your clinician as part of your ongoing clinical care.

If you have been taking pramipexole, you will be given advice on how to safely reduce your dose over several week unless your clinical team confirms that you can continue taking it after the end of the study (see p11 for end of study arrangements).

When should I take study medication?

The study medication should be taken by mouth once a day, usually at night. Your study doctor will provide further advice on this when prescribing the medication.

What if I miss a dose?

If you have missed a dose of study medication, but remember within 12 hours of the time you should have taken the dose, take that dose straightaway. If you remember more than 12 hours later, do not take anything until your next dose. Never take a double dose to make up for a forgotten tablet dose.

If you miss a dose whilst your dose is being increased (for example, in weeks 1 to 4), please continue on your current dose until you have achieved 3 days in a row of the required dose before you move up to the next dose.

What if I take too much medication?

If at any stage during the study you think or know that you may have taken too much medication you should contact emergency services if you need this, but also contact a study doctor using the contact information on your patient safety card. They will be able to advise you what to do.

Stopping medication early and withdrawing

If you decide at any time that you want to stop taking study medication before the end of the study, or if your clinical team recommend that you should stop early, then you will have to gradually reduce the dose of medication that you are on until you can safely stop taking it.

You must never suddenly stop taking the study medication.

Your clinical team will tell you how to safely reduce and stop the medication over several weeks to allow them to monitor your health as you do this. You should also keep completing the online questionnaires and RA telephone calls so that your safety can be monitored. When you have 2 weeks in a row when you haven't taken any drug, you will have a final study call. You will also need to return any bottles of medication (whether they are empty or they have tablets left in them) by either sending them in the mail in the returns envelope provided or by handing them over to the study team. Neither you nor your clinical team will know which treatment group you were allocated to, unless there is a clinical need to know this information.

Even though you aren't taking study medication you can still continue to complete the online questionnaires and receive RA telephone calls so the study team can continue to follow you until the end of the study. We would still value this information from you. You can decide whether or not you want to do this. If not, you will be withdrawn completely from the study but we will use any anonymised information that we have collected about you up until that point. We may ask you why you have chosen to withdraw, but you don't have to give a reason.

Study Diary

Completing the diary each day will help you keep track of information for the RA telephone calls. The RAs will be keeping a record of this information. The diary will be returned at the end of the study as it needs to be stored with the rest of the study documents, but we will not be reading it as the information will have already been collected. If you lose your diary, please let the RA know so they can send you a new one.

RA Telephone Calls

The study RAs will be calling you regularly (weekly until week 12 and then every 4 weeks until the end of the study). They will arrange the calls at a time to suit you, ideally on a similar day or time for each call. You will be able to call, text or email if you need to rearrange for a different time.

Each phone call, you will be asked questions which will include:

- what dose you are taking and if you have missed any doses
- potential side effects you may have been experiencing and any symptoms you may have had
- any other medication, including the mood stabilising medication, you are taking and if there have been any changes
- if you have experienced any impulse control symptoms, manic symptoms, depressive symptoms or suicidal thoughts.
- if you have any problems with the online questionnaires, if you have missed any questions etc.

Completing your diary will help you remember the answers to these questions.

In your first phone call of stage 2 and then at week 12, you will also be asked to complete a couple of questionnaires over the phone. These should take around 5 to 10 minutes each.

- **Young Mania Self-Rating Scale (YMRS):**
  This is used to find out about any manic symptoms you might have experienced.
- **Montgomery-Asberg Depression Rating Scale (MADRS) and Quick Inventory of Depressive Symptomatology – Clinician Rated (QIDS-C)**
  These are used to find out about any depressive symptoms you might have experienced.

Please note, the study RAs who call you are collecting information for the study but are not medically trained. It is important that you also discuss any symptoms or feelings with your doctor.

Returning Medication

Any unused medication from the previous prescription and/or any empty bottles will be collected for return (see p4 for further information about medication returns). Any readings that cause concern will be reported back to your clinical team.

True Colours: Online Questionnaires

You will need to continue completing your online questionnaires using the True Colours system. You can access this on a computer, smartphone or tablet. The system will remind you when you need to complete these. Paper versions can be made available if necessary.

Your answers will be used to find to out more about the effect of the study medication, and also reviewed for your safety. If the answers to your questions raise any concerns, this will be passed on to your clinical team, to help them review if your dose of medication should be changed.

What are the questionnaires?

You will be asked to complete three following questionnaires every week during stage 2. This should take around 10-15 minutes of your time in total.

- **Quick Inventory of Depressive Symptomatology – Self Report (QIDS-SR)**
  This is used to find out about any depressive symptoms you might have experienced.
- **Altman Self-rating Mania Scale (ASRM)**
  This is used to find out about any manic symptoms you might have experienced.
- **Generalised Anxiety Disorder 7 (GAD-7)**
  This is used to find out about any anxiety you might have experienced.

Before you take any study medication at the start of the stage 2, and then at weeks 6, 12, and every 4 weeks after that, you will also be asked to complete some of the additional following questionnaires. These will take you around 5 minutes each, and they don't all need to be completed at the same time – you can come back to them over a couple of days.

- **Snaith–Hamilton Pleasure Scale (SHAPS)**
  This is used to find out about depressive symptoms you might have experienced related to ability to experience pleasure.
- **Work and Social Adjustment Scale (WSAS)**
  This is used to find out about how you feel you function in day to day life.
- **Treatment Satisfaction Questionnaire for Medication (TSQM)**
  This is used to find out about how you feel about the medication you are taking.
- **Questionnaire for Impulsive-Compulsive Disorders in Parkinson's Disease – Rating Scale (QUIP-RS)**
  This is used to find out about any impulse control symptoms you might have experienced (such as compulsive gambling, buying, eating or sexual behaviour).
- **EuroQoL 5 Dimension 5 Level (EQ-5D-5L), ICEpop CAPability measure for Adults (ICECAP-A), Oxford CAPabilities questionnaire-Mental Health (OxCAP-MH), The Health Economics Questionnaire (HEQ)**
  These are used to find out about how you feel about your quality of life.

End of Study Information

Week 46: you will be asked to think about whether you want to stay on study medication after the study has ended. You will not know what medication you are taking at this point. This gives your clinical team more time to make arrangements for the drug to be available for you after the end of the study if necessary.

- ***Week 48:*** you will not automatically find out if you have been taking the active study drug pramipexole, or the dummy placebo drug during the study. However if you would like to continue taking pramipexole outside of the study the local team can make arrangements to find out what you have been taking. In this case:
    - If you have been taking **placebo**, you will not need to continue taking it and can stop when your clinical team tells you to do so.
    - If you have been receiving placebo but would like to try taking pramipexole outside of the trial you will be able to discuss this option with your clinician as part of your ongoing clinical care.
    - If you have been taking **pramipexole** and you have said you **do** want to continue taking it after the end of the study, your clinical team will have time to try to make arrangements for this medication available to you outside of the study. Enough study medication will be prescribed until the end of **week 52**.
    - If you have been taking pramipexole and then decide you do **not** want to continue taking it after the end of the study, your clinical team will discuss with you how to safely reduce your medication over several weeks before you can stop.

If at week 48 you do not want to continue taking pramipexole outside of the study, the team will not find out for you at this stage what you have been taking during the study. Arrangements can be made for you to find out once the study has completely finished if you would like to know this.

Never stop your study medication suddenly. Always speak to your study doctor or local clinical team first about how to do stop safely. They will help you to safely reduce and stop the medication over several weeks and they will monitor your health as you do this.

You will continue to be cared for by your usual clinic team after the end of the study. Please note that pramipexole can’t be provided by the study after week 52. Taking pramipexole outside of the study will depend on your doctor being able to make arrangements for this locally.

At the end of the study, if you are a female of childbearing potential you will also be asked for a urine sample to confirm that you are not currently pregnant.

Side Effects
As with any medicine, the medication used in this study may cause side effects.

**Some <u>very common</u> side effects of pramipexole that have been reported are:**
• Drowsiness/sleepiness<br>• Dizziness<br>• Abnormal or uncontrolled involuntary movements<br>• Nausea
**<u>Other commonly</u> reported side effects of pramipexole include:**
• Trouble sleeping (insomnia), hallucinations, abnormal dreams, confusion, impulse control behavioural symptoms<br>• Headache<br>• Visual impairment such as double or blurred vision, or reduced clarity<br>• Low blood pressure<br>• Constipation<br>• Vomiting<br>• Fatigue (tiredness)<br>• Fluid accumulation (oedema)<br>• Weight loss<br>• Decreased appetite
**<u>Other uncommon</u> <u>or rarely reported</u> side effects of pramipexole include:**
• Dopamine agonist withdrawal syndrome: this includes a lack of interest/enthusiasm, anxiety, depression, fatigue, sweating and pain<br>• Pneumonia<br>• Psychiatric symptoms such as compulsive shopping, pathological gambling, restlessness, hyper sexuality, delusion, libido disorder (increased or decreased), paranoia, delirium, binge or excessive eating, mania<br>• Sudden onset of sleep<br>• Memory loss (amnesia)<br>• Muscle spasms<br>• Fainting<br>• Heart failure<br>• Difficulty breathing<br>• Hiccups<br>• Skin hypersensitivity (rash/itch)

If you experience any of these side effects it is important that you let your doctor know straight away. The study doctor can talk to you more about what these mean if you are unsure about any of them. The side effects experienced may only be temporary as you adjust your dose but it is important to discuss these so that they can be monitored.

Driving
Taking pramipexole can have a major influence on the ability to drive and use machines. It can make you feel drowsy or sleepy and have episodes of suddenly falling asleep. If you experience this at any time whilst you are taking the study medication, you **must not** drive or

take part in any thing where not being alert might put you at risk of serious injury or death (like operating machines). You should tell your doctor if this occurs.

Further Supporting Information

The information in this section was also included in the information sheet you were given at the start of stage 1. We ask that you read through this again to make sure you are still happy and understand what taking part in stage 2 will mean for you.

Pregnancy

If you do become pregnant during the course of the study, you must tell your doctor **immediately** so appropriate action can be discussed. We will also ask you to consent to your clinical team following your pregnancy until completion.

Expenses and payments

As a thank you for taking part in the study, you will be provided with gift vouchers of £25 by post at weeks 12, 36 and 52.

What happens when the research study stops?

At the end of the study you will continue with your standard clinical care. This may include taking pramipexole if this has been arranged by your local clinician.

We hope that the results of this study will help us say if pramipexole is beneficial for patients with BD. You will be able to find out about the study results from the Northern Centre for Mood Disorders' website and through Bipolar UK. We will send you a copy of study results if you agree to this.

What are the benefits of taking part?

We cannot promise the study will help you directly but the information we get from this study may help to improve the treatment for people with treatment resistant bipolar depression. If you want to find out more about taking part in research studies you can visit the NHS Choices website www.nhs.uk.

What are the possible disadvantages or risks of taking part?

We want you to be safe in this study at all times, but all medical treatments carry some risk. Pramipexole is used by thousands of NHS patients with Parkinson's Disease or Restless Legs Syndrome, and there are some known side effects.

If you react badly to the drug your doctor will be able to change your medication and treat you to try to alleviate your symptoms. If they need to find out which treatment you are taking (pramipexole or placebo), this information is available in case of an emergency.

What will happen if I don't want to carry on with the study?

You can withdraw from the study medication and carry on completing the online questionnaires, or withdraw from the study completely. You can withdraw at any time for any reason, without giving a reason. You will be fully cared for and supported in line with your hospital's standard practice.

We will ask if you are happy for us to record why you decided to withdraw. If you withdraw from the study, we will keep and use the information about you that we have already obtained.

You must never suddenly stop taking the study medication.

What if there is a problem?
If you have a concern about any aspect of this study, you can speak to a member of the study team (this could be at your hospital or clinic, or one of the RAs) who will do their best to answer your questions. Further contact details are included at the end of this information sheet. If you are still unhappy and want to raise your concerns with someone who is not directly involved in your care, you can contact <site to localise with local details such as PALS phone number and email address>

If the unlikely event that you are harmed during the study and this is due to someone's negligence (they were careless) you may have grounds for legal action and compensation, but you may need to meet your own legal costs. NHS Indemnity does not offer no-fault compensation (for harm that is not anyone's fault).

The Newcastle Clinical Trials Unit, part of Newcastle University, are managing the study on behalf of the study NHS sponsor. Newcastle University also have insurance arrangements in place to cover Newcastle University staff involved in designing and managing the PAX-BD study.

What will happen to the results of the research study?
- The results will be written in medical journals and presented in meetings to other doctors, nurses, researchers and patients.
- A report will be written for the study funder and put on their website.
- All study data that is published will be anonymous. Your identity will always be protected.
- The results will be available at the end of the study through publications, in the wider press and directly to patient BD groups e.g. via Bipolar UK.
- Fully anonymised data may be made available to other researchers both within and outside the UK to help inform other research studies.

Will my taking part in this study be kept confidential?
Yes. All of the information collected will be entered on computers that are kept secure and password protected.
- You will be given a unique identification number instead of writing your name on study documents. Staff at your hospital/clinic will be able to link this number back to you using your date of birth, name and NHS number.
- Information with your ID number will only be shared with researchers on the study team, both in and outside of the UK.
- The Sponsor, Cumbria, Northumberland, Tyne and Wear NHS Foundation Trust (CNTW):
  - We will use your contact details for the RA telephone calls
  - Pharmacy will have access to your contact details for posting your study medication
- Your local study doctor will be contacting you about your study medication and your health.
- Your email address will be used for sending the True Colours reminders.
- Your name and address will be provided to Royal Mail so that your study medication can be posted to you

- Your contact details will never be shared with anyone else outside of the study.
- You will not be named in any results, reports or anything on our website.
- Very occasionally, information might be given during the study that we would have a legal obligation to pass on to others (for instance information which suggested you or others were at risk of harm). In this case, confidentiality would be broken so that we could pass this information to the relevant people. You would be informed of this.
- Site staff will have access to your information during the study to organise trial visits as well as for ongoing safety. At the end of the study, all trial information will be kept in a secure storage area (this is called archiving) for at least 5 years. This makes sure any queries about the running of the study have been answered. All information will be held securely to make sure we protect your confidentiality, after which it will be safely destroyed.
- We will ask your permission for a copy of the completed consent form to be sent securely to the Newcastle Clinical Trials Unit (NCTU). This is so that the NCTU team can carry out planned checks of completed forms. This is optional and you can write on the consent form if you agree to this or not.

Who is organising and funding the research?
The central study doctor (also known as the 'Chief Investigator') is Professor Hamish McAllister-Williams, a Consultant Psychiatrist and Professor of Affective Disorders. He is based in Newcastle upon Tyne. The study team also include senior doctors and nurses, university experts in research studies and members of the public.

Study sponsor: Cumbria, Northumberland, Tyne and Wear NHS Foundation Trust. PAX-BD is managed by the Newcastle Clinical Trials Unit on behalf of the sponsor.
Study funder: the National Institute for Health Research (NIHR) Health Technology Assessment (HTA) programme (reference 16/154/01). This body is funded by the UK government to carry out research for the benefit of the NHS and its patients.

Up to 40 trusts will be taking part in the PAX-BD study. Each trust will have a study doctor, called an Investigator. The investigator for your trust is ____________________

Who has reviewed the study?
The funder reviewed the study plan as part of the application for funding. All research in the NHS is also looked at by an independent group of people, called a Research Ethics Committee, to protect your interests. This study has been reviewed and given favourable opinion by North East - Newcastle & North Tyneside 2 Research Ethics Committee.

Patients and carers, including from a Bipolar Support Group, have also been involved in deciding how to do PAX-BD. We also asked a group of patients with BD to look at the information sheets to make sure they are presented in a clear way, are easy to understand, and include important information. We also have patients on the different study committees who help oversee the running of the study.

Who is providing the study drug?
The study sponsor pharmacy will be sending the study medication (pramipexole or placebo) to PAX-BD participants in the post. They are based in Newcastle upon Tyne. A company called ModePharma has provided the study drug and made the placebo to match for this study.

What if relevant new information becomes available?

If, during the course of the study, new information becomes available that is relevant to you, we will tell you about it and discuss whether you should or would like to withdraw from the study. If it is better for you to withdraw, you can do this without giving a reason. This will not affect the care that you receive.

Further information and contact details

If you have any further questions or would like any further information about the study or the rights of participants, please feel free to contact the people below, or visit the PAX-BD website http://www.mood-disorders.co.uk/PAX-BD

They are also who you or your doctor should contact in the event of an emergency, if your study participation is in any way involved.

[LOCAL CONTACT DETAILS]

Thank you for your continuing interest in the PAX-BD study.

Further Information about the Optional Telephone Interviews

We would like to hear about peoples' experiences of taking part in PAX-BD. We want to speak to as many people as we can, but we will not be able to speak to everyone involved in the study. This is separate to the main study, so you do **not** have to agree to be interviewed. Please indicate on the study consent form whether or not you would like to be contacted for an interview. If we do contact you, a RA will get in touch to arrange a convenient date and time to do the interview.

- The interview will be over the telephone.
- The interview will last about 30 minutes, no longer than an hour (depending on how much you have to say). It will be conducted by a RA.
- We will ask your permission to audio-record the conversation so that the interviewer can talk to you without having to make too many notes.

How will the interview be used?
The conversation will be written out in full (transcribed) and used by the researchers to improve this and future studies. Your name and any personal details will be removed from the written version to make sure you cannot be identified (anonymised). We hope to publish the results of the study in scientific journals, which may include anonymised quotes from the conversations.

Will what I tell you be kept confidential?
- Any recordings of your voice and transcripts (written out version) will be kept securely on a secure password protected database at Cumbria, Northumberland, Tyne and Wear NHS Foundation Trust and University of Nottingham.
- The recordings will be shared with a professional transcription company to be written out in full.
- All recordings will be deleted at the end of the study.
- We will use a number to identify you instead of your name (we call this pseudo-anonymised). We will remove anything else that could identify you (anonymised).
- The anonymised transcripts will be kept to help with future research. We may want to use quotes from the transcripts but they will not include your name.
- Information about you will be looked at by people directly involved in the study, as well as by people who are checking it is running as it should.

Data Protection and Transparency Information

Cumbria, Northumberland, Tyne and Wear NHS Foundation Trust (CNTW) is the sponsor for this study based in the United Kingdom and will act as the “data controller” for this study. **They are responsible for looking after your information and using it properly.**

This study is managed on behalf of the sponsor by the Newcastle Clinical Trials Unit who will act as the “data processor”. As data processor, this means that we are responsible for processing personal data on behalf of a controller. We will be using information from you in order to undertake this study, and will keep identifiable information about you for 5 years after the study has finished.

TrueColours will act as a data processor for the study and the information that you enter on to the online platform will be stored securely on servers of the University of Oxford. This information will be transferred securely to the Sponsor and study researchers who will analyse your data together with the data of all the other participants. This data will be provided to the researchers in a pseudo-anonymised form, will be held securely and kept confidential. The information that you provide will be held for 5 years after the study has finished.

Royal Mail will act as a data controller for this study. The sponsor will provide Royal Mail with your name and the address you have provided to allow Royal Mail to deliver your study medication to you. Royal Mail has internal data retention policies which cover secure destruction of all information in compliance with their legal and regulatory obligations. Data provided to Royal Mail will be stored for a maximum of 13 months and 1 day before being destroyed. Royal Mail Group’s policy is to only retain information for as long as it is required for the purpose or purposes for which they use it.

Your rights to access, change or move your information are limited, as we need to manage your information in specific ways in order for the research to be reliable and accurate. If you withdraw from the study, we will keep the information about you that we have already obtained. To safeguard your rights, we will use the least amount of personally-identifiable information possible.

You can find out more about how your information is used at
www.cntw.nhs.uk/about/research/

To find out more about research and general use of patient information please refer to the Health Research Authority Website https://www.hra.nhs.uk/information-about-patients/

The local study team at your hospital/clinic and the central Research Assistants (RAs) will collect information from you and/or your medical records for this research study in accordance with our instructions.

The local study team and RAs will use your name, NHS number and contacts details to contact you about the research study, and make sure that relevant information about the study is recorded for your care, and to oversee the quality of the study. Individuals from the sponsor, Newcastle Clinical Trials Unit and regulatory organisations may look at your medical and research records to check the accuracy of the research study. The local study team will pass these details to the sponsor or the Newcastle Clinical Trials Unit along with information

collected from you and/or your medical records. The only people at sponsor or the Newcastle Clinical Trials Unit who will have access to information that identifies you will be people who need to contact you or audit the data collection process. The people who analyse the information will not be able to identify you and will not be able to find out your name, NHS number or contact details.

When you agree to take part in a research study, the information about your health and care may be provided to researchers running other research studies in this organisation and in other organisations. These organisations may be universities, NHS organisations or companies involved in health and care research in this country or abroad. Your information will only be used by organisations and researchers to conduct research in accordance with the UK Policy Framework for Health and Social Care Research.

This information will not identify you and will not be combined with other information in a way that could identify you. The information will only be used for the purpose of health and care research, and cannot be used to contact you or to affect your care. It will not be used to make decisions about future services available to you, such as insurance.

If you join the study, identifiable information about you will be stored and processed at your local hospital/clinic, the sponsor's Trust and Newcastle Clinical Trials Unit. All information which is collected about you during the course of the research will be stored in secure and locked offices, on a password-protected database and on secure Trust and University servers.
