# Supplementary material for: Study protocol for a randomised placebo-controlled trial of pramipexole in addition to mood stabilisers for patients with treatment resistant bipolar depression (the PAX-BD study)
Source: BMC Psychiatry. 2021 Jul 5;21:334. doi: 10.1186/s12888-021-03322-y (PMC8256234; doi:10.1186/s12888-021-03322-y)
Supplement: Supplementary file 6 — Additional file 6. [file 12888_2021_3322_MOESM6_ESM.pdf]

Newcastle University

PAX BD
Pramipexole Trial for Bipolar Depression

Trust headed paper

A randomised, double-blind, placebo controlled trial of pramipexole in addition to mood stabilisers for patients with treatment resistant bipolar depression (PAX-BD).

Informed Consent Form – Stage 2

V6.0 11/09/2020

CONFIDENTIAL ONCE COMPLETED

Site Number: ________________ **Participant Number:** ________________

Principal Investigator: ________________________________

Please INITIAL the boxes if you agree:

1.	I have read and understood the PAX-BD Randomisation Information Sheet version_____, dated ___________ and have had the opportunity to ask questions.	
2.	I understand that I do not have to take part in this study. I know that I can withdraw at any time and do not have to give a reason. I know that this will not affect my standard medical care or legal rights. I understand that if I withdraw from the study, the information collected from me until that point will be retained and used.	
3.	I understand I must not stop taking my trial medication suddenly. I know that if I want to stop taking the medication that I should discuss this with my doctor so I can do this safely.	
4.	I understand that parts of my medical records and data collected during the study may be looked at by responsible people. I give my permission for these people to have access to my medical records.<br><br>This includes people from the Newcastle Clinical Trials Unit, Newcastle University, study Sponsor, regulatory authorities and local NHS Trusts where it is relevant to my taking part in research.	
5.	I understand that the information I provide on TrueColours will be kept confidential, will be stored securely and will be used by study researchers in a pseudo-anonymised form.	
6.	I understand that any personal information collected about me for the study will be kept confidential and not be made public. I understand that the information collected about me will be used to support other research in the future, and may be shared anonymously with other researchers both within and outside the UK. I understand I will not be directly identified in the published results.	
7.	I understand that my personal data (including name, address, telephone number, email address, date of birth and gender) will be stored by responsible people at Cumbria, Northumberland, Tyne and Wear NHS Foundation Trust for the purposes of the study. I give permission for these people to store this information until the end of the study when it will be destroyed.	

8.	I understand that my GP and care team will be informed that I am taking part in this PAX-BD study.	
9.	I understand that the information provided in this study is being managed by the Newcastle Clinical Trials Unit.	
10.	I agree to the information provided and this signed consent form being stored for 5 years after the end of the trial.	
11.	I understand that my name and address will be provided to Royal Mail so that trial medication can be posted to me throughout my participation in the study.	
12.	**I agree to take part in the PAX-BD trial.**	

For female participants of childbearing potential

13.	I understand that if I am of childbearing potential, I will need to provide three urine sample(s) to make sure that I am not pregnant. I understand that this is for safety reasons.	
14.	I understand that I will have to use the forms of contraception that have been explained to me, if sexually active.	
15.	I understand that if I become pregnant I consent to allow the research team to follow my pregnancy to completion.	

STOP AND CHECK Please make sure you have <u>initialled</u> the boxes if you agree.

Optional for ALL participants

16.	I understand that a copy of this consent form will be submitted to Newcastle Clinical Trials Unit for the purposes of central monitoring and will be destroyed following a documented check of the form. I give permission for these individuals to receive a copy of this form.	
17.	I agree to participate in the qualitative phone interviews.	
18.	I understand that an audio-recording will be made of my qualitative interview.	
19.	I understand that the audio-recordings will be shared with a professional transcription service provider for the purposes of transcribing the interviews into text.	
20.	I understand that data from my audio-recorded interviews being transferred to and retained by Cumbria, Northumberland, Tyne and Wear NHS Foundation Trust and University of Nottingham for research purposes, now and in the future. I understand that this may include anonymised quotations.	
21.	I would like a summary of the results to be sent to me when the study has finished.	

Name of participant ____________ Signature ____________ Date ____________

Name of person taking consent ____________ Signature ____________ Date ____________

When completed - 1 copy for patient, 1 original copy for Investigator Site File and 1 copy for hospital records.
