# Supplementary material for: Study protocol for a randomised placebo-controlled trial of pramipexole in addition to mood stabilisers for patients with treatment resistant bipolar depression (the PAX-BD study)
Source: BMC Psychiatry. 2021 Jul 5;21:334. doi: 10.1186/s12888-021-03322-y (PMC8256234; doi:10.1186/s12888-021-03322-y)
Supplement: Supplementary file 7 — Additional file 7. [file 12888_2021_3322_MOESM7_ESM.pdf]

Appendix 7 – PAX-BD Participant Withdrawal Form V1.0 16/05/2019

To be printed on local headed paper

The PAX-BD Study

WITHDRAWAL FORM

Study ID Number: _______________

Principal Investigator Name: ______________________________

We understand that you would like to withdraw from the PAX-BD study. The study team will be happy to talk this through with you and answer any questions you have. Please remember that if you wish to stop taking study medication this does not mean you need to withdraw from the study – indeed it is of great value to continue to follow patients up who are no longer on study medication.

If you do wish to withdraw from the study entirely, ideally we would like to contact you for a final telephone interview. We might not be able to contact everyone, but if we do, one of the Research Assistants will call you to arrange a convenient date and time to do the interview.

Withdrawing from this study will not affect your usual care in any way. You can still contact your clinical team at the hospital as normal. ***Please note:*** *if you are withdrawing due to a serious adverse event, the research team are required to continue follow-up until the event has been resolved.*

Please initial the boxes that apply to you

1) I wish to withdraw from **the PAX-BD study.** I understand my data will be used up until the point that I withdrew. ☐

If you consented to be contacted for **telephone interview:**

2) I **no longer wish** to be contacted about a telephone interview ☐

3) I **am still happy** to be contacted about a telephone interview ☐

STOP and CHECK: Please make sure you have initialled the boxes and not ticked them.

Reason for withdrawal: *(please remember you* ***do not have to*** *give us a reason if you don't want to):*

____

____

Participant

Signed .... Print Name.. Date ....

Investigator

Signed .... Print Name.. Date ....

(Original form to be stored in the site file, 1 copy given to the participant and 1 copy to be kept in participant medical notes.)
