# Supplementary material for: Study protocol for a randomised placebo-controlled trial of pramipexole in addition to mood stabilisers for patients with treatment resistant bipolar depression (the PAX-BD study)
Source: BMC Psychiatry. 2021 Jul 5;21:334. doi: 10.1186/s12888-021-03322-y (PMC8256234; doi:10.1186/s12888-021-03322-y)
Supplement: Supplementary file 8 — Additional file 8. [file 12888_2021_3322_MOESM8_ESM.pdf]

Newcastle University

PAX BD
Pramipexole Trial for Bipolar Depression

Trust headed paper

The PAX-BD Study

End of Study Participation Information Sheet
Withdrawal or Early Completion of Trial Medication

Thank you for taking part in the PAX-BD study. Research can ask a lot of questions and we appreciate your time and effort. It is only with your help that we can carry out research to try and find new treatments for patients with bipolar disorder.

You have either withdrawn from the study completely or you have stopped taking the study medication early. This information sheet should answer any questions you may have about your withdrawal from the study and your ongoing care.

This information sheet is split into three sections:

Section 1:	How you will safely come off the study medication	Page 2
Section 2:	The final assessments for you to complete	Page 2
Section 3:	Some further information including how and when you can find out the study results	Page 3

Section 1:

How do I stop taking the study medication?

Your doctor will tell you how to safely reduce and stop your study medication and this will happen over several weeks. They will check on your health to make sure this is done safely. Please **do not stop taking your medication or change your dose** without discussing this with your doctor first.

What if I miss a dose or take too much?

There is information about what to do if you miss a dose of medication or take too much medication in your participant diary. If you need to get in touch with your local study team please use the contact information at the end of this information sheet.

Section 2:

What are the final assessments that I need to complete?

During the weeks you are safely reducing and stopping your medication you should continue to:

- Complete the study participant diary
- Fill in your online questionnaires
- Take part in the telephone calls with the study team

These assessments will make sure your safety can be closed checked. After not taking any study medication for **2 weeks in a row** you will also have a final safety contact from your local clinical team. This can take place in your home, or at your usual clinic/hospital if you prefer.

What will happen during the final safety contact?

During the final safety contact we will:

- Arrange collection / to receive any empty medication bottles or leftover medication you may have.
- If relevant, ask you for a urine sample to carry out a pregnancy test.

Section 3:

What if I become pregnant?

Please tell you doctor straight away if you become pregnant at any time during the study, including this stage when your dose of medication is being gradually reduced.

Withdrawing from the study:

You can withdraw from the study at any time for any reason and you will continue to be cared for by your usual doctor and care team. You do not have to tell us why you withdrew but it can help us identify any areas we could make better.

If you withdraw from the study, we will keep and use the information about you that we have already collected. If you stop taking the study medication early you can still continue completing the online questionnaires and getting telephone calls from the study team. If not, you will be withdrawn completely from the study and we will only use any anonymised (we can't identify you) information that we have collected about you up until that point.

Will I get to find out what medication I received?

The team will not be able to find out which medication you have been taking while the study is still open. Arrangements can be made for you to find out once the study has completely finished.

Will my taking part in this study be kept confidential?

Yes, all of the information collected will be kept confidential.

- You will not be named in any results, reports or publications on our website.
- Very occasionally, information might be given during the study that we would have a legal obligation to pass on to others (for example information which suggested you or others were at risk of harm). In this case, confidentiality would be broken so that we could pass this information to the relevant people and you would be informed.
- At the end of the study, all trial information will be kept in a secure storage area (archived) for at least 5 years. All information will be held securely to make sure we protect your confidentiality, after which it will be safely destroyed.

What if I have any problems or concerns?

If you have a concern about any aspect of this study, please speak to your doctor or a member of the study team (this could be at your hospital, or one of the research assistants). If you want to raise your concerns with someone who is not directly involved in your care, you can get in touch <site to localise with local details such as PALS phone number and email address>

How can I find out the study results?

We hope this study will help identify if pramipexole is beneficial for patients with Bipolar Disorder. You will be able to find out about the study results from the Northern Centre for Mood Disorders' website and through Bipolar UK. Your local study team will also send you a summary of study results if you have consented to this. If you said no at the start of the study and have now changed your mind, please let a member of the study team know.

On behalf of the study team, thank you again for taking part in the PAX-BD study. For further information, or if you have any questions please contact your local study team:

LOCAL STUDY TEAM CONTACT DETAILS
