# Supplementary material for: Study protocol for a randomised placebo-controlled trial of pramipexole in addition to mood stabilisers for patients with treatment resistant bipolar depression (the PAX-BD study)
Source: BMC Psychiatry. 2021 Jul 5;21:334. doi: 10.1186/s12888-021-03322-y (PMC8256234; doi:10.1186/s12888-021-03322-y)
Supplement: Supplementary file 9 — Additional file 9. [file 12888_2021_3322_MOESM9_ESM.pdf]

Appendix 9 – PAX-BD Participant End of Trial Information Sheet – Trial Completion V2.0 27/07/2020

PAXBD
Pramipexole Trial for Bipolar Depression

Trust headed paper

The PAX-BD Study

End of Study Participation Information Sheet
Trial Completion

Thank you for taking part in the PAX-BD study. Research can ask a lot of questions and we appreciate your time and effort. It is only with your help that we can carry out research to try and find new treatments for patients with bipolar disorder.

As you come to the end of the study, you may have questions about how it will end and what this might mean for your care. This information sheet answers these questions.

This information sheet is split into three sections:

Section 1:	How you will safely come off the study medication	Page 2
Section 2:	The final assessments for you to complete	Page 3
Section 3:	Some further information including how and when you can find out the study results	Page 4

Section 1:

How will my time in the study end?

Towards the end of the study you will complete some final assessments and be asked whether you want to continue taking pramipexole once you have finished the study.

At week 46 your doctor will ask you to *think* about whether you want to continue taking pramipexole. At week 48 your doctor will then ask you if you *want* to continue taking pramipexole.

What if I want to continue taking Pramipexole?

If you want to continue taking pramipexole your doctor will find out if you have been taking the placebo or pramipexole and will let you know.

- If you have been taking the placebo you will not need to continue taking it and can stop when your doctor tells you to do so. If you would like to try pramipexole after the study you will be able to discuss this option with your doctor.
- If you have been taking pramipexole and want to continue after the study your doctor will look into the options for you to continue to do so. In the meantime, you will continue to take study pramipexole until the study finishes. If you change your mind please talk with your doctor **before** stopping your study medication or changing your dose. Stopping the medication suddenly can lead to very serious side effects.

What if I don't want to continue taking Pramipexole?

Your doctor will tell you how to safely reduce and stop your study medication and this will happen over several weeks. They will closely check your health to make sure this is done safely. Please **do not stop taking your medication or change your dose** without talking to your doctor first.

What if I miss a dose or take too much?

There is information about what to do if you miss a dose of medication or take too much medication in your participant diary. If you need to get in touch with your local study team please use the contact information at the end of this information sheet.

Section 2:

What are the final assessments that I need to complete?

During the final weeks of the study you should continue to:

- Complete the study participant diary
- Fill in your online questionnaires
- Take part in the telephone calls with the study team

These assessments will make sure your safety can be checked closely. You will also have a final safety contact from your local clinical team. This can take place in your home, or at your usual clinic/hospital if you prefer.

What will happen during the final safety contact?

During the final safety contact we will:

- Arrange collection / to receive any empty medication bottles or leftover medication you may have.
- If relevant, ask you for a urine sample to carry out a pregnancy test.

<u>Section 3:</u>

What if I become pregnant as the study ends?

You need to tell your study doctor straight away if you become pregnant at any time during the study, including this final stage when your dose of medication is being gradually reduced.

What will happen to my care once the study has finished?

You will continue to be cared for by your usual doctor and care team after the end of the study. If you want to continue taking or start taking pramipexole please speak to your usual doctor and care team to see if it is available.

What about expenses or payments?

As a thank you for taking part in the study, you will be given a gift voucher of £25 by post when you complete week 52.

Will my taking part in this study be kept confidential?

Yes, all of the information collected will be kept confidential.

- You will not be named in any results, reports or publications on our website.
- Very occasionally, information might be given during the study that we would have a legal obligation to pass on to others (for example information which suggested you or others were at risk of harm). In this case, confidentiality would be broken so that we could pass this information to the relevant people and you would be informed.
- At the end of the study, all trial information will be kept in a secure storage area for at least 5 years. All information will be held securely to make sure we protect your confidentiality, after which it will be safely destroyed.

What if I have any problems or concerns?

If you have a concern about any aspect of this study, please speak to your doctor or to a member of the study team (this could be at your hospital, or one of the research assistants). If you want to raise your concerns with someone who is not directly involved in your care, you can contact <site to localise with local details such as PALS phone number and email address>

How can I find out the study results?

We hope this study will help to find out if pramipexole is beneficial for patients with Bipolar Disorder. You will be able to find out about the study results from the <u>Northern Centre for Mood Disorders' website and through Bipolar UK.</u> Your local study team will also send you a summary of study results if you have consented to this. If you said no at the start of the study and have changed your mind, please let a member of the study team know.

On behalf of the study team, thank you again for taking part in the PAX-BD study. For further information, or if you have any questions please contact your local study team:

LOCAL STUDY TEAM CONTACT DETAILS
