# Supplementary material for: Study protocol for a randomised placebo-controlled trial of pramipexole in addition to mood stabilisers for patients with treatment resistant bipolar depression (the PAX-BD study)
Source: BMC Psychiatry. 2021 Jul 5;21:334. doi: 10.1186/s12888-021-03322-y (PMC8256234; doi:10.1186/s12888-021-03322-y)
Supplement: Supplementary file 10 — Additional file 10. [file 12888_2021_3322_MOESM10_ESM.pdf]

PAX BD

Pramipexole Trial for Bipolar Depression

Participant Diary

Version 3.0 10/12/2019

Participant Study Number:	
Please enter the date you started taking your study medication:	_ _/ _ _/ 20_ _

Instructions to Complete Patient Diary

- This diary is for you to complete. It will help you remember information that we need to collect from you during the study.
- Please have this diary at hand for Research Assistant (RA) phone calls.
- Please also remember to log into the online True Colours system to fill in the questionnaires when prompted to.

EudraCT Number: 2018-2869-18
Study Sponsored by Cumbria, Northumberland, Tyne and Wear NHS Foundation Trust

Contents

My Study Information....................3

Dose Information....................4

Missed Doses....................6

Dose Log....................7

Side Effects....................19

Any other medication....................21

Study Drug Instructions....................23

Study Schedule....................24

My Study Information

You may find it helpful to use this section to note down the names and contact information of people involved in this study. If you have any concerns or questions about the study you can speak to a member of the study team below.

My study doctor(s):

Research Assistant(s):

Other members of my study team (e.g. Clinical Study Officers):

Study website: https://mood-disorders.co.uk/PAX-BD/

At the end of week 12, please return this diary back to a member of the research team. Thank you.

Dose Information

ALWAYS REFER TO YOUR CURRENT STUDY DOSING SCHEDULE. YOUR DOSE WILL CHANGE DURING THE STUDY.

Your dosing schedule will tell you exactly what dose you need to take. Always try and take your study medication at the same time every evening (unless you are advised otherwise by your doctor).

Once you know the dose you need to take, the table below tells you how to make up your dose correctly. It is important that you **follow these instructions only to make your daily dose**, as there may not be enough tablets if you use other combinations.

There will be 56 tablets in each bottle of medication.

1mg tablet = **BLUE** label 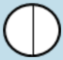
0.25mg tablet = **YELLOW** label 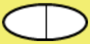

Dose to be taken ONCE daily	Number of 1mg tablets to take (BLUE label)	Number of 0.25mg tablets to take (YELLOW label)	Total number of tablets to be taken per day
0.25mg	0	1	1
0.5mg	0	2	2
0.75mg	0	3	3
1.0mg	1	0	1
1.25mg	1	1	2
1.5mg	1	2	3
1.75mg	1	3	4
2.0mg	2	0	2
2.25mg	2	1	3
2.5mg	2	2	4

Missed Doses

If you have missed a dose, but remember within 12 hours of the time you should have taken the dose, take that dose straightaway. If you remember more than 12 hours later, do not take anything until your next dose. **Never take a double dose to make up for a forgotten tablet dose.**

If you have **missed a dose whilst your dose is being increased or flexibly dosed,** you must keep taking your current dose until you have completed 3 days in a row of this dose before you can change the dose. Please see an example below.

Week	Day	Date	Dose taken (mg)	Number tablets taken		Missed Dose (✓)	Reason for Missed/Incorrect Dose or Comments
				1mg	0.25mg		
2	8	03/03/2019	0·75	0	3		
	9	04/03/2019	0	0	0	✓	Forgot to take tablets
	10	05/03/2019	0·75	0	3		3 consecutive days at 0.75mg before increasing to 1.0mg.
	11	06/03/2019	0·75	0	3		
	12	07/03/2019	0·75	0	3		
	13	08/03/2019	1·0	1	0		
	14	09/03/2019	1·0	1	0		

A space is provided under each table for you to provide any additional information about why you may have missed your dose or that may be relevant to your ability to take the tablets for the week. Please see an example below.

Please provide any information you think may be relevant to your ability to take tablets this week
I missed a dose of my medication on day 25 because I was very ill with vomiting and could not keep any food down.

Dose Log

Please use the table below to record the dates that you take your study medication, and what you **actually** took that day including the size and number of tablets. Please also record if you missed a dose for any reason.

Week	Day	Date	Dose taken (mg)	Number tablets taken		Missed Dose (✓)	Reason for Missed/Incorrect Dose or Comments
				1mg	0.25mg		
**Example**		01/01/2019	1.25	1	1		
1	1						
	2						
	3						
	4						
	5						
	6						
	7						

Week	Day	Date	Dose taken (mg)	Number tablets taken		Missed Dose (✓)	Reason for Missed/Incorrect Dose or Comments
				1mg	0.25mg		
2	8						
	9						
	10						
	11						
	12						
	13						
	14						

Please provide any information you think may be relevant to your ability to take tablets this week
If you have started using a new medication bottle this week, please give the date started:

Week	Day	Date	Dose taken (mg)	Number tablets taken		Missed Dose (✓)	Reason for Missed/Incorrect Dose or Comments
				1mg	0.25mg		
3	15						
	16						
	17						
	18						
	19						
	20						
	21						

Please provide any information you think may be relevant to your ability to take tablets this week
If you have started using a new medication bottle this week, please give the date started:

Week	Day	Date	Dose taken (mg)	Number tablets taken		Missed Dose (✓)	Reason for Missed/Incorrect Dose or Comments
				1mg	0.25mg		
4	22						
	23						
	24						
	25						
	26						
	27						
	28						

Please provide any information you think may be relevant to your ability to take tablets this week
If you have started using a new medication bottle this week, please give the date started:

Week	Day	Date	Dose taken (mg)	Number tablets taken		Missed Dose (✓)	Reason for Missed/Incorrect Dose or Comments
				1mg	0.25mg		
5	29						
	30						
	31						
	32						
	33						
	34						
	35						

Please provide any information you think may be relevant to your ability to take tablets this week
If you have started using a new medication bottle this week, please give the date started:

Week	Day	Date	Dose taken (mg)	Number tablets taken		Missed Dose (✓)	Reason for Missed/Incorrect Dose or Comments
				1mg	0.25mg		
6	36						*Remember to start your new bottles of medication today*
	37						
	38						
	39						
	40						
	41						
	42						

Please provide any information you think may be relevant to your ability to take tablets this week
If you have started using a new medication bottle this week, please give the date started:

Week	Day	Date	Dose taken (mg)	Number tablets taken		Missed Dose (✓)	Reason for Missed/Incorrect Dose or Comments
				1mg	0.25mg		
7	43						
	44						
	45						
	46						
	47						
	48						
	49						

Please provide any information you think may be relevant to your ability to take tablets this week
If you have started using a new medication bottle this week, please give the date started:

Week	Day	Date	Dose taken (mg)	Number tablets taken		Missed Dose (✓)	Reason for Missed/Incorrect Dose or Comments
				1mg	0.25mg		
8	50						
	51						
	52						
	53						
	54						
	55						
	56						

Please provide any information you think may be relevant to your ability to take tablets this week
If you have started using a new medication bottle this week, please give the date started:

Week	Day	Date	Dose taken (mg)	Number tablets taken		Missed Dose (✓)	Reason for Missed/Incorrect Dose or Comments
				1mg	0.25mg		
9	57						
	58						
	59						
	60						
	61						
	62						
	63						

Please provide any information you think may be relevant to your ability to take tablets this week
If you have started using a new medication bottle this week, please give the date started:

Week	Day	Date	Dose taken (mg)	Number tablets taken		Missed Dose (✓)	Reason for Missed/Incorrect Dose or Comments
				1mg	0.25mg		
10	64						
	65						
	66						
	67						
	68						
	69						
	70						

Please provide any information you think may be relevant to your ability to take tablets this week
If you have started using a new medication bottle this week, please give the date started:

Week	Day	Date	Dose taken (mg)	Number tablets taken		Missed Dose (✓)	Reason for Missed/Incorrect Dose or Comments
				1mg	0.25mg		
11	71						
	72						
	73						
	74						
	75						
	76						
	77						

Please provide any information you think may be relevant to your ability to take tablets this week
If you have started using a new medication bottle this week, please give the date started:

Week	Day	Date	Dose taken (mg)	Number tablets taken		Missed Dose (✓)	Reason for Missed/Incorrect Dose or Comments
				1mg	0.25mg		
12	78						
	79						
	80						
	81						
	82						
	83						
	84						

Please provide any information you think may be relevant to your ability to take tablets this week
If you have started using a new medication bottle this week, please give the date started:

Side Effects

If you experience any symptoms whilst taking the study medication, please enter the details in the table below. Research Assistants and Clinical Study officers will ask you about these during their calls and visits.

**Symptom**	**Description of symptoms**	**Start Date**	**End Date**
e.g. Headache	e.g. pressure pain at the back of the head	01/02/2019	02/02/2019

Symptom	Description of symptoms	Start Date	End Date

Any other medication

Please enter details in the table below if you take any medication (either on prescription or over the counter). Please also record any herbal medications or recreational drugs you have taken. Research Assistants will ask you about these during their calls.

Drug Name	Dose Taken	How often do you take it?	Why do you take it?	What date did you start taking it?	What date did you stop taking it?
e.g. Paracetamol	1g	once daily	Headache	02/01/2019	04/01/2019

Drug Name	Dose Taken	How often do you take it?	Why do you take it?	What date did you start taking it?	What date did you stop taking it?

Study Drug Instructions

Study medication will arrive by Royal Mail (signed for) delivery. Week 1 starts when you start taking the study medication. Please ensure that you have completed all of your 'baseline assessments' before starting to take the study medication. Please see your study information sheet for further information, or ask the RA when they call if you are unsure.

There will be **seven** lots of study medication sent to you during the study. Your study medication will usually arrive around a week before it is needed, as per the table below:

Study Medication will be posted at these times:	To be taken in these weeks:
Before week 1	Weeks 1-5 (5 weeks)
By the start of week 5	Weeks 6-12 (7 weeks)
By the start of week 12	Weeks 13-20 (8 weeks)
By the start of week 20	Weeks 21-28 (8 weeks)
By the start of week 28	Weeks 29-36 (8 weeks)
By the start of week 36	Weeks 37-44 (8 weeks)
By the start of week 44	Weeks 45-52 (8 weeks)

We will let you know when your study medication is on its way. Please let the RA or your doctor know if your next batch of medication has not arrived. You should start taking the new medication in the weeks listed above, even if there are tablets left in the old bottles.

Please keep all bottles (whether they are empty or they have medication left) and hand them over when you next see a member of the study team.

You should keep your medication in a safe place and out of the reach of children.

Study Schedule

- **Weeks 1-4**: your dose will be started at the lowest level of 0.25mg daily. This will increase by 0.25mg every 3 days until you have reached a maximum of 2.5mg, or your maximum tolerated dose. If you are unsure about increasing your dose you can continue on your current dose for a further 3 days before deciding to increase it.
- **Weeks 5-12:** you will continue on the same dose you were taking at the end of week 4 (unless otherwise advised by your study doctor).
- **Week 13-48:** during this period, your study doctor will review how you respond and tolerate the medication and discuss with you whether to increase or decrease your daily dose (up to a maximum of 2.5mg daily).
- **Week 46:** you will be asked to think about whether you might want to stay on the study medication at the end of the study.
- **Week 48:** You will not automatically find out if you have been taking the active study drug pramipexole, or the dummy placebo drug during the study. However if you would like to continue taking pramipexole outside of the study, you can confirm this to your local team at this visit and they can then make arrangements to find out what you have been taking. In this case:
    - If you have been taking placebo, you will not need to continue taking it and can stop when your clinical team tells you to do so.
    - If you have been receiving placebo but would like to try taking pramipexole outside of the trial you will be able to discuss this option with your clinician as part of your ongoing clinical care.
    - If you have been taking pramipexole and you have said you do wish to continue taking it after the end of the study, your clinical team will have time to try to make arrangements for this medication available to you outside of the study. Enough study medication will be prescribed until the end of week 52. Your final study visit will be at **week 52**.
    - If you have been taking pramipexole and then decide you do not wish to continue taking it after the end of the study, your clinical team will

discuss with you how to safely reduce your medication over several weeks before you can stop.

If at week 48 you do not wish to continue taking pramipexole outside of the study, the team will not find out for you at this stage what you have been taking during the study. Arrangements can be made for you to find out once the study has completely finished if you would like to know this. Your clinical team will advise you how to safely reduce your trial medication over several weeks before you can stop. Your final study visit will be at week 52 or when you have been trial medication free for 2 weeks (whichever is later).

Thank you for completing this participant diary.
Please hand this diary back to a member of the study team at your next study visit.
