# Supplementary material for: Study protocol for a randomised placebo-controlled trial of pramipexole in addition to mood stabilisers for patients with treatment resistant bipolar depression (the PAX-BD study)
Source: BMC Psychiatry. 2021 Jul 5;21:334. doi: 10.1186/s12888-021-03322-y (PMC8256234; doi:10.1186/s12888-021-03322-y)
Supplement: Supplementary file 11 — Additional file 11. [file 12888_2021_3322_MOESM11_ESM.pdf]

PAX BD

Pramipexole Trial for Bipolar Depression

Participant Diary

Version 3.0 10/12/2019

Participant Study Number:	
Please enter the date you started taking your study medication:	__/__/ 20__

Instructions to Complete Patient Diary

- This diary is for you to complete. It will help you remember information that we need to collect from you during the study.
- Please have this diary at hand for Research Assistant (RA) phone calls.
- Please also remember to log into the online True Colours system to fill in the questionnaires when prompted to.

EudraCT Number: 2018-2869-18
Study Sponsored by Cumbria, Northumberland, Tyne and Wear NHS Foundation Trust

Contents

My Study Information 3

Dose Information 4

Missed Doses 6

Dose Log 7

Side Effects 49

Any other medication 52

Study Drug Instructions 55

Study Schedule 56

My Study Information

You may find it helpful to use this section to note down the names and contact information of people involved in this study.

My study doctor(s):

Research Assistant(s):

Other members of my study team (e.g. Clinical Study Officers):

Study website: https://mood-disorders.co.uk/PAX-BD/

At the end of the study, please return this diary back to a member of the research team. Thank you.

Dose Information

ALWAYS REFER TO YOUR CURRENT STUDY DOSING SCHEDULE. YOUR DOSE WILL CHANGE DURING THE STUDY.

Your dosing schedule will tell you exactly what dose you need to take. Always try and take your study medication at the same time every evening (unless you are advised otherwise by your doctor).

Once you know the dose you need to take, the table below tells you how to make up your dose correctly. It is important that you **follow these instructions only to make your daily dose**, as there may not be enough tablets if you use other combinations.

There will be 56 tablets in each bottle of medication.

1mg tablet = **BLUE** label 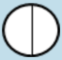
0.25mg tablet = **YELLOW** label 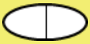

Dose to be taken ONCE daily	Number of 1mg tablets to take (BLUE label)	Number of 0.25mg tablets to take (YELLOW label)	Total number of tablets to be taken per day
0.25mg	0	1	1
0.5mg	0	2	2
0.75mg	0	3	3
1.0mg	1	0	1
1.25mg	1	1	2
1.5mg	1	2	3
1.75mg	1	3	4
2.0mg	2	0	2
2.25mg	2	1	3
2.5mg	2	2	4

Missed Doses

If you have missed a dose, but remember within 12 hours of the time you should have taken the dose, take that dose straightaway. If you remember more than 12 hours later, do not take anything until your next dose. **Never take a double dose to make up for a forgotten tablet dose.**

If you have **missed a dose whilst your dose is being increased or flexibly dosed,** you must keep taking your current dose until you have completed 3 days in a row of this dose before you can change the dose. Please see an example below.

Week	Day	Date	Dose taken (mg)	Number tablets taken		Missed Dose (✓)	Reason for Missed/Incorrect Dose or Comments
				1mg	0.25mg		
2	8	03/03/2019	0·75	0	3		
	9	04/03/2019	0	0	0	✓	Forgot to take tablets
	10	05/03/2019	0·75	0	3		3 consecutive days at 0.75mg before increasing to 1.0mg.
	11	06/03/2019	0·75	0	3		
	12	07/03/2019	0·75	0	3		
	13	08/03/2019	1·0	1	0		
	14	09/03/2019	1·0	1	0		

A space is provided under each table for you to provide any additional information about why you may have missed your dose or that may be relevant to your ability to take the tablets for the week. Please see an example below.

Please provide any information you think may be relevant to your ability to take tablets this week
I missed a dose of my medication on day 25 because I was very ill with vomiting and could not keep any food down.

Dose Log

Please use the table below to record the dates that you take your study medication, and what you **actually** took that day including the size and number of tablets. Please also record if you missed a dose for any reason.

Week	Day	Date	Dose taken (mg)	Number tablets taken		Missed Dose (✓)	Reason for Missed/Incorrect Dose or Comments
				1mg	0.25mg		
Wk 13	85						*Remember to start your new bottles of medication today*
	86						
	87						
	88						
	89						
	90						
	91						

Please provide any information you think may be relevant to your ability to take tablets this week
If you have started using a new medication bottle this week, please give the date started:

Week	Day	Date	Dose taken (mg)	Number tablets taken		Missed Dose (✓)	Reason for Missed/Incorrect Dose or Comments
				1mg	0.25mg		
Wk 14	92						
	93						
	94						
	95						
	96						
	97						
	98						

Please provide any information you think may be relevant to your ability to take tablets this week
If you have started using a new medication bottle this week, please give the date started:

Week	Day	Date	Dose taken (mg)	Number tablets taken		Missed Dose (✓)	Reason for Missed/Incorrect Dose or Comments
				1mg	0.25mg		
Wk 15	99						
	100						
	101						
	102						
	103						
	104						
	105						

Please provide any information you think may be relevant to your ability to take tablets this week
If you have started using a new medication bottle this week, please give the date started:

Week	Day	Date	Dose taken (mg)	Number tablets taken		Missed Dose (✓)	Reason for Missed/Incorrect Dose or Comments
				1mg	0.25mg		
Wk 16	106						
	107						
	108						
	109						
	110						
	111						
	112						

Please provide any information you think may be relevant to your ability to take tablets this week
If you have started using a new medication bottle this week, please give the date started:

Week	Day	Date	Dose taken (mg)	Number tablets taken		Missed Dose (✓)	Reason for Missed/Incorrect Dose or Comments
				1mg	0.25mg		
Wk 17	113						
	114						
	115						
	116						
	117						
	118						
	119						

Please provide any information you think may be relevant to your ability to take tablets this week
If you have started using a new medication bottle this week, please give the date started:

Week	Day	Date	Dose taken (mg)	Number tablets taken		Missed Dose (✓)	Reason for Missed/Incorrect Dose or Comments
				1mg	0.25mg		
Wk 18	120						
	121						
	122						
	123						
	124						
	125						
	126						

Please provide any information you think may be relevant to your ability to take tablets this week
If you have started using a new medication bottle this week, please give the date started:

Week	Day	Date	Dose taken (mg)	Number tablets taken		Missed Dose (✓)	Reason for Missed/Incorrect Dose or Comments
				1mg	0.25mg		
Wk 19	127						
	128						
	129						
	130						
	131						
	132						
	133						

Please provide any information you think may be relevant to your ability to take tablets this week
If you have started using a new medication bottle this week, please give the date started:

Week	Day	Date	Dose taken (mg)	Number tablets taken		Missed Dose (✓)	Reason for Missed/Incorrect Dose or Comments
				1mg	0.25mg		
Wk 20	134						
	135						
	136						
	137						
	138						
	139						
	140						

Please provide any information you think may be relevant to your ability to take tablets this week
If you have started using a new medication bottle this week, please give the date started:

Week	Day	Date	Dose taken (mg)	Number tablets taken		Missed Dose (✓)	Reason for Missed/Incorrect Dose or Comments
				1mg	0.25mg		
Wk 21	141						*Remember to start your new bottles of medication today*
	142						
	143						
	144						
	145						
	146						
	147						

Please provide any information you think may be relevant to your ability to take tablets this week
If you have started using a new medication bottle this week, please give the date started:

Week	Day	Date	Dose taken (mg)	Number tablets taken		Missed Dose (✓)	Reason for Missed/Incorrect Dose or Comments
				1mg	0.25mg		
Wk 22	148						
	149						
	150						
	151						
	152						
	153						
	154						

Please provide any information you think may be relevant to your ability to take tablets this week
If you have started using a new medication bottle this week, please give the date started:

Week	Day	Date	Dose taken (mg)	Number tablets taken		Missed Dose (✓)	Reason for Missed/Incorrect Dose or Comments
				1mg	0.25mg		
Wk 23	155						
	156						
	157						
	158						
	159						
	160						
	161						

Please provide any information you think may be relevant to your ability to take tablets this week
If you have started using a new medication bottle this week, please give the date started:

Week	Day	Date	Dose taken (mg)	Number tablets taken		Missed Dose (✓)	Reason for Missed/Incorrect Dose or Comments
				1mg	0.25mg		
Wk 24	162						
	163						
	164						
	165						
	166						
	167						
	168						

Please provide any information you think may be relevant to your ability to take tablets this week
If you have started using a new medication bottle this week, please give the date started:

Week	Day	Date	Dose taken (mg)	Number tablets taken		Missed Dose (✓)	Reason for Missed/Incorrect Dose or Comments
				1mg	0.25mg		
Wk 25	169						
	170						
	171						
	172						
	173						
	174						
	175						

Please provide any information you think may be relevant to your ability to take tablets this week
If you have started using a new medication bottle this week, please give the date started:

Week	Day	Date	Dose taken (mg)	Number tablets taken		Missed Dose (✓)	Reason for Missed/Incorrect Dose or Comments
				1mg	0.25mg		
Wk 26	176						
	177						
	178						
	179						
	180						
	181						
	182						

Please provide any information you think may be relevant to your ability to take tablets this week
If you have started using a new medication bottle this week, please give the date started:

Week	Day	Date	Dose taken (mg)	Number tablets taken		Missed Dose (✓)	Reason for Missed/Incorrect Dose or Comments
				1mg	0.25mg		
Wk 27	183						
	184						
	185						
	186						
	187						
	188						
	189						

Please provide any information you think may be relevant to your ability to take tablets this week
If you have started using a new medication bottle this week, please give the date started:

Week	Day	Date	Dose taken (mg)	Number tablets taken		Missed Dose (✓)	Reason for Missed/Incorrect Dose or Comments
				1mg	0.25mg		
Wk 28	190						
	191						
	192						
	193						
	194						
	195						
	196						

Please provide any information you think may be relevant to your ability to take tablets this week
If you have started using a new medication bottle this week, please give the date started:

Week	Day	Date	Dose taken (mg)	Number tablets taken		Missed Dose (✓)	Reason for Missed/Incorrect Dose or Comments
				1mg	0.25mg		
Wk 29	197						*Remember to start your new bottles of medication today*
	198						
	199						
	200						
	201						
	202						
	203						

Please provide any information you think may be relevant to your ability to take tablets this week
If you have started using a new medication bottle this week, please give the date started:

Week	Day	Date	Dose taken (mg)	Number tablets taken		Missed Dose (✓)	Reason for Missed/Incorrect Dose or Comments
				1mg	0.25mg		
Wk 30	204						
	205						
	206						
	207						
	208						
	209						
	210						

Please provide any information you think may be relevant to your ability to take tablets this week
If you have started using a new medication bottle this week, please give the date started:

Week	Day	Date	Dose taken (mg)	Number tablets taken		Missed Dose (✓)	Reason for Missed/Incorrect Dose or Comments
				1mg	0.25mg		
Wk 31	211						
	212						
	213						
	214						
	215						
	216						
	217						

Please provide any information you think may be relevant to your ability to take tablets this week
If you have started using a new medication bottle this week, please give the date started:

Week	Day	Date	Dose taken (mg)	Number tablets taken		Missed Dose (✓)	Reason for Missed/Incorrect Dose or Comments
				1mg	0.25mg		
Wk 32	218						
	219						
	220						
	221						
	222						
	223						
	224						

Please provide any information you think may be relevant to your ability to take tablets this week
If you have started using a new medication bottle this week, please give the date started:

Week	Day	Date	Dose taken (mg)	Number tablets taken		Missed Dose (✓)	Reason for Missed/Incorrect Dose or Comments
				1mg	0.25mg		
Wk 33	225						
	226						
	227						
	228						
	229						
	230						
	231						

Please provide any information you think may be relevant to your ability to take tablets this week
If you have started using a new medication bottle this week, please give the date started:

Week	Day	Date	Dose taken (mg)	Number tablets taken		Missed Dose (✓)	Reason for Missed/Incorrect Dose or Comments
				1mg	0.25mg		
Wk 34	232						
	233						
	234						
	235						
	236						
	237						
	238						

Please provide any information you think may be relevant to your ability to take tablets this week
If you have started using a new medication bottle this week, please give the date started:

Week	Day	Date	Dose taken (mg)	Number tablets taken		Missed Dose (✓)	Reason for Missed/Incorrect Dose or Comments
				1mg	0.25mg		
Wk 35	239						
	240						
	241						
	242						
	243						
	244						
	245						

Please provide any information you think may be relevant to your ability to take tablets this week
If you have started using a new medication bottle this week, please give the date started:

Week	Day	Date	Dose taken (mg)	Number tablets taken		Missed Dose (✓)	Reason for Missed/Incorrect Dose or Comments
				1mg	0.25mg		
Wk 36	246						
	247						
	248						
	249						
	250						
	251						
	252						

Please provide any information you think may be relevant to your ability to take tablets this week
If you have started using a new medication bottle this week, please give the date started:

Week	Day	Date	Dose taken (mg)	Number tablets taken		Missed Dose (✓)	Reason for Missed/Incorrect Dose or Comments
				1mg	0.25mg		
Wk 37	253						*Remember to start your new bottles of medication today*
	254						
	255						
	256						
	257						
	258						
	259						

Please provide any information you think may be relevant to your ability to take tablets this week
If you have started using a new medication bottle this week, please give the date started:

Week	Day	Date	Dose taken (mg)	Number tablets taken		Missed Dose (✓)	Reason for Missed/Incorrect Dose or Comments
				1mg	0.25mg		
Wk 38	260						
	261						
	262						
	263						
	264						
	265						
	266						

Please provide any information you think may be relevant to your ability to take tablets this week
If you have started using a new medication bottle this week, please give the date started:

Week	Day	Date	Dose taken (mg)	Number tablets taken		Missed Dose (✓)	Reason for Missed/Incorrect Dose or Comments
				1mg	0.25mg		
Wk 39	267						
	268						
	269						
	270						
	271						
	272						
	273						

Please provide any information you think may be relevant to your ability to take tablets this week
If you have started using a new medication bottle this week, please give the date started:

Week	Day	Date	Dose taken (mg)	Number tablets taken		Missed Dose (✓)	Reason for Missed/Incorrect Dose or Comments
				1mg	0.25mg		
Wk 40	274						
	275						
	276						
	277						
	278						
	279						
	280						

Please provide any information you think may be relevant to your ability to take tablets this week
If you have started using a new medication bottle this week, please give the date started:

Week	Day	Date	Dose taken (mg)	Number tablets taken		Missed Dose (✓)	Reason for Missed/Incorrect Dose or Comments
				1mg	0.25mg		
Wk 41	281						
	282						
	283						
	284						
	285						
	286						
	287						

Please provide any information you think may be relevant to your ability to take tablets this week
If you have started using a new medication bottle this week, please give the date started:

Week	Day	Date	Dose taken (mg)	Number tablets taken		Missed Dose (✓)	Reason for Missed/Incorrect Dose or Comments
				1mg	0.25mg		
Wk 42	288						
	289						
	290						
	291						
	292						
	293						
	294						

Please provide any information you think may be relevant to your ability to take tablets this week
If you have started using a new medication bottle this week, please give the date started:

Week	Day	Date	Dose taken (mg)	Number tablets taken		Missed Dose (✓)	Reason for Missed/Incorrect Dose or Comments
				1mg	0.25mg		
Wk 43	295						
	296						
	297						
	298						
	299						
	300						
	301						

Please provide any information you think may be relevant to your ability to take tablets this week
If you have started using a new medication bottle this week, please give the date started:

Week	Day	Date	Dose taken (mg)	Number tablets taken		Missed Dose (✓)	Reason for Missed/Incorrect Dose or Comments
				1mg	0.25mg		
Wk 44	302						
	303						
	304						
	305						
	306						
	307						
	308						

Please provide any information you think may be relevant to your ability to take tablets this week
If you have started using a new medication bottle this week, please give the date started:

Week	Day	Date	Dose taken (mg)	Number tablets taken		Missed Dose (✓)	Reason for Missed/Incorrect Dose or Comments
				1mg	0.25mg		
Wk 45	309						*Remember to start your new bottles of medication today*
	310						
	311						
	312						
	313						
	314						
	315						

Please provide any information you think may be relevant to your ability to take tablets this week
If you have started using a new medication bottle this week, please give the date started:

Week	Day	Date	Dose taken (mg)	Number tablets taken		Missed Dose (✓)	Reason for Missed/Incorrect Dose or Comments
				1mg	0.25mg		
Wk 46	316						
	317						
	318						
	319						
	320						
	321						
	322						

Please provide any information you think may be relevant to your ability to take tablets this week
If you have started using a new medication bottle this week, please give the date started:

Week	Day	Date	Dose taken (mg)	Number tablets taken		Missed Dose (✓)	Reason for Missed/Incorrect Dose or Comments
				1mg	0.25mg		
Wk 47	323						
	324						
	325						
	326						
	327						
	328						
	329						

Please provide any information you think may be relevant to your ability to take tablets this week
If you have started using a new medication bottle this week, please give the date started:

Week	Day	Date	Dose taken (mg)	Number tablets taken		Missed Dose (✓)	Reason for Missed/Incorrect Dose or Comments
				1mg	0.25mg		
Wk 48	330						
	331						
	332						
	333						
	334						
	335						
	336						

Please provide any information you think may be relevant to your ability to take tablets this week
If you have started using a new medication bottle this week, please give the date started:

Week	Day	Date	Dose taken (mg)	Number tablets taken		Missed Dose (✓)	Reason for Missed/Incorrect Dose or Comments
				1mg	0.25mg		
Wk 49	337						
	338						
	339						
	340						
	341						
	342						
	343						

Please provide any information you think may be relevant to your ability to take tablets this week
If you have started using a new medication bottle this week, please give the date started:

Week	Day	Date	Dose taken (mg)	Number tablets taken		Missed Dose (✓)	Reason for Missed/Incorrect Dose or Comments
				1mg	0.25mg		
Wk 50	344						
	345						
	346						
	347						
	348						
	349						
	350						

Please provide any information you think may be relevant to your ability to take tablets this week
If you have started using a new medication bottle this week, please give the date started:

Week	Day	Date	Dose taken (mg)	Number tablets taken		Missed Dose (✓)	Reason for Missed/Incorrect Dose or Comments
				1mg	0.25mg		
Wk 51	351						
	352						
	353						
	354						
	355						
	356						
	357						

Please provide any information you think may be relevant to your ability to take tablets this week
If you have started using a new medication bottle this week, please give the date started:

Week	Day	Date	Dose taken (mg)	Number tablets taken		Missed Dose (✓)	Reason for Missed/Incorrect Dose or Comments
				1mg	0.25mg		
Wk 52	358						
	359						
	360						
	361						
	362						
	363						
	364						

Please provide any information you think may be relevant to your ability to take tablets this week
If you have started using a new medication bottle this week, please give the date started:

Week	Day	Date	Dose taken (mg)	Number tablets taken		Missed Dose (✓)	Reason for Missed/Incorrect Dose or Comments
				1mg	0.25mg		
Wk 53	365						
	366						
	367						
	368						
	369						
	370						
	371						

Please provide any information you think may be relevant to your ability to take tablets this week
If you have started using a new medication bottle this week, please give the date started:

Week	Day	Date	Dose taken (mg)	Number tablets taken		Missed Dose (✓)	Reason for Missed/Incorrect Dose or Comments
				1mg	0.25mg		
Wk 54	372						
	373						
	374						
	375						
	376						
	377						
	378						

Please provide any information you think may be relevant to your ability to take tablets this week
If you have started using a new medication bottle this week, please give the date started:

Side Effects

If you experience any symptoms whilst taking the study medication, please enter the details in the table below. Research Assistants and Clinical Study officers will ask you about these during their calls and visits.

Symptom	Description of symptoms	Start Date	End Date
e.g. Headache	e.g. pressure pain at the back of the head	01/02/2019	02/02/2019

Symptom	Description of symptoms	Start Date	End Date

Symptom	Description of symptoms	Start Date	End Date

Any other medication

Please enter details in the table below if you take any medication (either on prescription or over the counter). Please also record any herbal medications or recreational drugs you have taken. Research Assistants will ask you about these during their calls.

Drug Name	Dose Taken	How often do you take it?	Why do you take it?	What date did you start taking it?	What date did you stop taking it?
e.g. Paracetamol	1g	once daily	Headache	02/01/2019	04/01/2019

Drug Name	Dose Taken	How often do you take it?	Why do you take it?	What date did you start taking it?	What date did you stop taking it?

Drug Name	Dose Taken	How often do you take it?	Why do you take it?	What date did you start taking it?	What date did you stop taking it?

Study Drug Instructions

Study medication will arrive by Royal Mail (signed for) delivery. Week 1 starts when you start taking the study medication. Please ensure that you have completed all of your 'baseline assessments' before starting to take the study medication. Please see your study information sheet for further information, or ask the RA when they call if you are unsure.

There will be **seven** lots of study medication sent to you during the study. Your study medication will usually arrive around a week before it is needed, as per the table below:

Study Medication will be posted at these times:	To be taken in these weeks:
Before week 1	Weeks 1-5 (5 weeks)
By the start of week 5	Weeks 6-12 (7 weeks)
By the start of week 12	Weeks 13-20 (8 weeks)
By the start of week 20	Weeks 21-28 (8 weeks)
By the start of week 28	Weeks 29-36 (8 weeks)
By the start of week 36	Weeks 37-44 (8 weeks)
By the start of week 44	Weeks 45-52 (8 weeks)

We will let you know when your study medication is on its way. Please let the RA or your doctor know if your next batch of medication has not arrived. You should start taking the new medication in the weeks listed above, even if there are tablets left in the old bottles.

Please keep all bottles (whether they are empty or they have medication left) and hand them over when you next see a member of the study team.

You should keep your medication in a safe place and out of the reach of children.

Study Schedule

- **Weeks 1-4**: your dose will be started at the lowest level of 0.25mg daily. This will increase by 0.25mg every 3 days until you have reached a maximum of 2.5mg, or your maximum tolerated dose. If you are unsure about increasing your dose you can continue on your current dose for a further 3 days before deciding to increase it.
- **Weeks 5-12:** you will continue on the same dose you were taking at the end of week 4 (unless otherwise advised by your study doctor).
- **Week 13-48:** during this period, your study doctor will review how you respond and tolerate the medication and discuss with you whether to increase or decrease your daily dose (up to a maximum of 2.5mg daily).
- **Week 46:** you will be asked to think about whether you might want to stay on the study medication at the end of the study.
- **Week 48:** You will not automatically find out if you have been taking the active study drug pramipexole, or the dummy placebo drug during the study. However if you would like to continue taking pramipexole outside of the study, you can confirm this to your local team at this visit and they can then make arrangements to find out what you have been taking. In this case:
    - If you have been taking placebo, you will not need to continue taking it and can stop when your clinical team tells you to do so.
    - If you have been receiving placebo but would like to try taking pramipexole outside of the trial you will be able to discuss this option with your clinician as part of your ongoing clinical care.
    - If you have been taking pramipexole and you have said you do wish to continue taking it after the end of the study, your clinical team will have time to try to make arrangements for this medication available to you outside of the study. Enough study medication will be prescribed until the end of week 52. Your final study visit will be at **week 52**.
    - If you have been taking pramipexole and then decide you do not wish to continue taking it after the end of the study, your clinical team will

discuss with you how to safely reduce your medication over several weeks before you can stop.

If at week 48 you do not wish to continue taking pramipexole outside of the study, the team will not find out for you at this stage what you have been taking during the study. Arrangements can be made for you to find out once the study has completely finished if you would like to know this. Your clinical team will advise you how to safely reduce your trial medication over several weeks before you can stop. Your final study visit will be at week 52 or when you have been trial medication free for 2 weeks (whichever is later).

Thank you for completing this participant diary.
Please hand this diary back to a member of the study team at your next study visit.
