# Supplementary material for: Study protocol for a randomised placebo-controlled trial of pramipexole in addition to mood stabilisers for patients with treatment resistant bipolar depression (the PAX-BD study)
Source: BMC Psychiatry. 2021 Jul 5;21:334. doi: 10.1186/s12888-021-03322-y (PMC8256234; doi:10.1186/s12888-021-03322-y)
Supplement: Supplementary file 12 — Additional file 12. [file 12888_2021_3322_MOESM12_ESM.pdf]

Appendix 12 – PAX-BD Qualitative Interview Topic Guides

Interview Topics for Service Users who are randomised to the trial and reach week 12

Please remember to separately ask about withdrawal from antipsychotic drugs and introduction of mood stabiliser before randomisation, randomisation itself and titration of drug after randomisation, attitude to remaining on experimental drug after randomisation for 12 weeks and then 12 months). Use your judgment about this depending on what seems relevant to the participant.

- How did you hear about the research study?
  - Who first mentioned the research study to you?
- What was your initial reaction to the invitation to participate?
  - How did you feel about taking part?
- What factors impacted on your decision to take part in the study?
  - Why did you decide to join the research study?
  - Was there anything in particular that persuaded you to join the study?
  - Prompts: health (current symptoms)
  - Prompts: attitudes to research (opportunity to access new services or treatment approaches (withdrawal from antipsychotics, introduction of mood stabiliser, new drug , monitoring using digital methods (True colours), treatment and monitoring by specialists in bipolar disorder or depression, altruism, risk taking, hope)
    Prompts: engaging the patient (active promotion and marketing, trust, clarity of trial process)
- Were there also concerns that you had about taking part?
  - Were there any factors that put you off?
  - Prompts: health (expression of depression symptoms, fear of symptom exacerbation/risk of trial to mental health, concern over side effects of mood stabiliser or pramipexole , concern over withdrawal of antipsychotic drug, concern over length of time before improvement in depression with slow titration, lack of choice over use of other treatments especially if getting worse or not improving, vulnerability)
  - Prompts: attitudes to research (randomisation, randomisation to placebo, negative views about one of the treatment options, being experimented on, lack of choice, data security, medical model of management alone)
  - Prompts: engaging the patient (perceived stigma, challenges of understanding the trial, burden)
  - Prompts: practical issues (travel to the treatment centre, required time off work or from other commitments, availability of carers)
  - Did you complete the full cycle of treatment? Where did you get with the trial or have you got so far? Any incidents or concerns so far?
- Having received treatment through the study, can you tell me about your views on the treatment you received?
  - Are there any benefits that you have experienced?
  - Have there been any disadvantages/anything you disliked about the treatment?
  - How acceptable did you find the treatment?
- How did you find completing the self-rating scales and questionnaires as part of the study?
  - Any advantages / disadvantages you can think of (e.g. time commitment, technical or use issues)?

    - Do you have any thoughts/feedback/suggestions?
- What was your experience of the Research Assistant contacts during the study?
    - How did you find your interactions with the trial Research Assistant?
    - How did you view their role, as part of this study?
    - Did you have any issues with the scheduled contacts?
    - Did you have to contact them outside of the Study schedule? If yes, how did you find this?

Interview Topics for Service Users who either 1) withdraw/are withdrawn during the pre-randomisation phase, or who are not randomised OR, 2) are randomised but stop taking trial medication prior to week 12

- How did you hear about the research study?
    - Who first mentioned the research study to you?
- What was your initial reaction to the invitation to participate?
    - How did you feel about taking part?
- What factors impacted on your decision to not take part in the study?
    - Why did you decide not to join the research study?
    - Prompts: health (expression of depression symptoms, fear of symptom exacerbation/risk of trial to mental health, concern over side effects of pramipexole or mood stabiliser drugs, concern over withdrawal of antipsychotic drug, concern over length of time before improvement in depression with slow titration, lack of choice over use of other treatments especially if getting worse or not improving, vulnerability)
    - Prompts: attitudes to research (randomisation, negative views about one of the treatment options, randomisation to placebo, being experimented on, lack of choice, data security, medical model of management alone)
    - Prompts: engaging the patient (perceived stigma, challenges of understanding the trial, burden)
    - Prompts: practical issues (travel to the treatment centre, required time off work or from other commitments, availability of carers)
- Were there any positives to taking part in the study for you?
    - Was there anything that could have persuaded you to take part in the study?
    - Prompts: health (current symptoms)
    - Prompts: attitudes to research (opportunity to access new services or treatment approaches (withdrawal from antipsychotics, introduction of mood stabiliser, new drug , monitoring using digital methods (True colours), treatment and monitoring by specialists in bipolar disorder or depression, altruism, risk taking, hope)
      Prompts: engaging the patient (active promotion and marketing, trust, clarity of trial process)
    - If there are positives in taking part, what swayed them not to take part? What might have made a difference to this decision?
- How did you find completing the self-rating scales and questionnaires as part of the study?
    - Any advantages / disadvantages you can think of (e.g. time commitment, technical or use issues)?
    - Do you have any thoughts/feedback/suggestions?
- What was your experience of the Research Assistant contacts during the study?
    - How did you find your interactions with the trial Research Assistant?

    - How did you view their role, as part of this study?
    - Did you have any issues with the scheduled contacts?
    - Did you have to contact them outside of the Study schedule? If yes, how did you find this?

Interview Topics for Principal Investigators and professionals at participating sites open for 4 months

- What is your current role in relation to the service?
    - Prompt: prior experience with service users with depression
- What is your current role in relation to the study?
    - Prompt: prior experience with recruitment into a research trial
- What has been your experience of recruiting participants to the trial?
    - How well has it gone?
    - Are some people more likely to be interested? Why is this?
- Have there been any specific barriers to recruitment – anything that people mention that puts them off?
    - Prompts: health (current symptoms, fear of symptom exacerbation, side effects of pramipexole or mood stabiliser, concern over withdrawal of antipsychotic drug, concern over length of time before improvement in depression with slow titration, lack of choice over use of other treatments especially if getting worse or not improving, self-stigma, vulnerability)
    - Prompts: attitudes to research (randomisation, negative views about one of the treatment options, randomisation to placebo, being experimented on, lack of choice, data security, medical model of management alone)
    - Prompts: engaging the patient (perceived stigma, challenges of communicating the trial, burden)
    - Prompts: complexity of the study, study processes
- Have there been any specific facilitators to recruitment – anything that people mention that encourages them to take part in the study?
    - Prompts: health (current symptoms)
    - Prompts: attitudes to research (opportunity to access new services or treatment approaches (withdrawal from antipsychotics, introduction of mood stabiliser, new drug , monitoring using digital methods (True colours), treatment and monitoring by specialists in bipolar disorder or depression, altruism, risk taking, hope)
    - Prompts: engaging the patient (active promotion and marketing, trust, clarity of trial process)
    - Prompts: support provided by the research team, local PI/Clinician guide.
- Are there any additional training or resources needed to support recruitment?
- Are there any other strategies that could support the recruitment of participants into the study?
- If there are positives in taking part, what swayed them personally to take part or not take part? What might have made a difference to this decision?
    - Prompts: gaining research experience, being able to report this in appraisal, to support Clinical Excellence Awards
    - Prompt: Gaining clinical experience using pramipexole, managing bipolar disorder.
    - Prompts: financial income to Trust for taking part
- How have you found using the Clinician Manual / Protocol?

- Did you have any issues, or areas of success, when using the manual?
- Do you have any specific feedback (positive and negative)?
- Do you have any suggestions or amendments you would welcome in future versions of the manual?
