# Supplementary material for: Study protocol for a randomised placebo-controlled trial of pramipexole in addition to mood stabilisers for patients with treatment resistant bipolar depression (the PAX-BD study)
Source: BMC Psychiatry. 2021 Jul 5;21:334. doi: 10.1186/s12888-021-03322-y (PMC8256234; doi:10.1186/s12888-021-03322-y)
Supplement: Supplementary file 13 — Additional file 13. [file 12888_2021_3322_MOESM13_ESM.pdf]

Appendix 13 – Substantial Amendment 7 for PAX-BD Protocol V6.0

In response to COVID-19, the following updates were made to the study protocol to allow delivery to continue during government and local restrictions, without compromising participant safety or study primary outcomes:

- Updated to allow the first approach of participants to be made by providing an invitation letter and study summary leaflet by post.
- Updated to allow pre-randomisation and randomisation consent discussions via telephone, teleconference or videoconference before the patient attends a shorter face to face clinic visit to complete the consent form.
- Updated to include completion of the Mini-International Neuropsychiatric Interview and Bipolar Demographics and Treatment Questionnaire over the phone or videoconference.
- Updated to allow screening visit Quick Inventory of Depressive Symptomology – Self Report (QIDS-SR) to be posted or emailed to participants, with responses being provided securely via email, telephone or videoconference. The questionnaire is also now available via the study website, allowing participants to access this in advance of a screening telephone call/videoconference. If it is not possible for the participant to review a copy of the QIDS-SR in advance (i.e. no internet access, paper copy lost in the post etc) and videoconference is also not an option (where the questionnaire could be displayed via screen share), then as a last resort the questionnaire can be completed over the phone with the questions read out to the participant by the site team.
- Updated to include additional methods for urine sample collection to complete pregnancy tests to confirm eligibility to the pre-randomisation and randomization phases, as well as the final participant safety assessment at the end of the study. Where it is not possible for the sample to be collected during an on-site visit, the sample pot will be sent out to the participant by post or given in advance (for example, when the participant attends to consent, at a standard clinic visit etc). The sample can then be collected at a standard clinic visit, a socially distanced door stop collection by site staff or participants can post samples back to the site teams themselves using a pre-paid envelope.
- Updated to include additional methods for providing participants with study diary and collection of completed diaries. Study diaries can be provided to participants either at a standard clinic visit, a socially distanced door stop collection by site staff or post. Completed copies will need to be returned by the participant while attending a standard clinic visit, a socially distanced door stop collection by site staff or a pre-paid envelope.
- Removed blood pressure and pulse measurements at weeks 0, 2, 6, 12, 24, 36 and 48. Symptoms of hypotension will continue to be monitored by Research Assistants during phone calls and reported as adverse events if present. Tolerability will also be

examined through an examination of adverse events. This was agreed by the Data Monitoring Committee.

- Updated to include additional methods of returning unused medication and empty bottles to the Cumbria, Northumberland, Tyne and Wear (CNTW) Pharmacy. These can be collected at a standard clinic visit, a socially distanced door stop collection by site staff or participants can return unused medication and empty bottles directly to CNTW themselves using a pre-paid envelope.
- Updated to include additional methods around completing the withdrawal form. This can be provided either at a standard clinic visit, by post or email.
- Updated to include additional methods of providing participants with the End of Study Information Sheet. This can be provided either at a standard clinic visit, by post or email.
